# Distribution and Health Risk Assessment of Triclosan and Other Typical Endocrine Disruptors in Honey

**DOI:** 10.3390/foods14122006

**Published:** 2025-06-06

**Authors:** Jianing Wang, Meiqi Gao, Hongmei Li, Xinyan Hou, Aijun Gong, Yanqiu Cao

**Affiliations:** 1 School of Chemistry and Biological Engineering, University of Science and Technology Beijing, Beijing 100083, China; wjn990516@163.com (J.W.); gaomq1211@163.com (M.G.); houxinyan12@163.com (X.H.); gongaijun@ustb.edu.cn (A.G.); 2 Division of Metrology in Chemistry, National Institute of Metrology, Beijing 100020, China; lihm@nim.accn

**Keywords:** typical endocrine disruptors, honey, high efficiency liquid chromatography, Ultrasonication-assisted dispersive liquid–liquid microextraction

## Abstract

Endocrine disruptors (EDCs) in food pose a significant threat to health. This study developed a method for detecting seven EDCs (triclosan (TCS), triclocarban (TCC), methyltriclosan (MTCS), methylparaben (MeP), propylparaben (PrP), bisphenol F (BPF), and 4-hydroxybenzophenone-3-ethylcarboxylate (4HBP)) in honey. The method combines ultrasonic-assisted dispersive liquid–liquid microextraction with high-performance liquid chromatography. It achieved a recovery rate of 89.70–102.2%, with an RSD value of 1.1–3.9%. Additionally, this study tested 47 honey samples from seven countries, revealing detection rates of TCS at 29.79%, TCC at 19.15%, BPF at 97.87%, 4HBP at 36.17%, MeP at 82.98%, and PrP at 80.85%. Among the 12 nectar sources, citrus flower nectar had the highest TCS detection rate, mother grass nectar had the highest TCC detection rate, and multi-flower nectar had the highest 4HBP detection rate. Moreover, imported honey samples showed higher levels of TCS, BPF, and MeP contamination compared to domestic samples. Honey stored in PET bottles contained the highest levels of EDCs. Finally, health risk assessments indicated that, while the risk for adults is lower, monitoring EDC contamination in food should be strengthened to ensure consumer safety.

## 1. Introduction

Endocrine disruptors currently comprise more than 800 different compounds that have been found in air, land, drinking water, plant and animal foods, consumer and personal care product, fuels, pharmaceuticals, and synthetic hormones [1,2,3]. There are several classes of chemicals that are categorized as typical endocrine disruptors. These chemicals can be categorized into several classes, including antimicrobial agents such as triclosan, which is commonly used in food and personal care products (PPCPs). The xenoestrogenic BPs are produced in industry. The ultraviolet filter benzophenones (BzPs) and PBs are substances used as preservatives. TCS and TCC are antimicrobial agents commonly used in many daily products. MTCS, a derivative of TCS, is a possible endocrine disruptor that is also currently reported. These chemicals, when in contact with the body for prolonged periods of time, can affect normal endocrine functions, such as affecting the reproductive system, the nervous system, the immune system, causing genetic mutations and carcinogenic effects [4]. TCS has endocrine disruptive capabilities; Gee, R. H. et al. demonstrated that TCS has estrogenic and androgenic activity [5]. In addition, exposure to TCS is associated with reproductive and developmental toxicity. The oral administration of TCS to pregnant mice (gestation days 1–16) has been shown to result in maternal and fetal toxicity, as evidenced by maternal mortality, reduced litter size, and reduced pup weights [6]. TCC may inhibit soluble epoxide hydrolase in vivo, leading to methemoglobinemia, upregulate gene expression associated with estrogen and androgen receptor responses, and disrupt neonatal birth weight, gestation, and body length [7]. There are few studies of BPF toxicity in vivo, and the main studies in the literature so far have been in rats and zebrafish. The study concluded that BPF has estrogenic, androgenic, and thyroid hormone activity. In 2007, Higashihara et al. [8] found that BPF triggered an increase in thyroid mass in male rats. A number of in vitro studies on the toxicity of BPF have concluded that BPF has estrogenic and antiandrogenic activity, and its endocrine-disrupting activity is comparable to that of BPA [9]. In addition, BPF may have genotoxicity, causing genetic damage by interfering with the DNA replication process [10]. Studies have shown that PBs have estrogenic activity [11], and BuP has the highest estrogenic potency of any substance in its class [12]. In animal studies, PBs exhibit weak estrogenic and thyrotoxic activities [13] as well as anti-androgenic properties [14]. Studies have shown that endocrine disruptors present in the aquatic environment can contaminate drinking water, soil, and aquatic organisms, etc., and then enter the human body through the food chain, thus causing adverse effects on human health. These endocrine disruptors can contaminate drinking water, soil, and aquatic organisms through the aquatic environment and then enter the human body via the food chain, causing adverse health effects.

Food contamination with endocrine disruptors (EDCs) can occur through various pathways, including water, air, and food, as well as during storage and processing, because contaminants are lipid-soluble and therefore easily accumulate in human tissues [15]. EDCs had been detected in several types of food samples. Xuedong Wang et al. [16] detected TCS in milk samples at 1.04 μg/kg. This widespread contamination has been documented in numerous food samples. Yao Li et al. [17] collected fish bile samples in the Yangtze River Basin, China, and detected four parabens and two antimicrobial agents. Methyl p-hydroxybenzoate (MeP) was detected in the range of 8.17–21.9 ng/mL, ethyl p-hydroxybenzoate (EtP) in the range of 0–31.6 ng/mL, propyl p-hydroxybenzoate (PrP) in the range of 2.19–112 ng/mL, and propyl 4-hydroxybenzoate (PrP) in the range of 2.19–112 ng/mL. Propyl p-hydroxybenzoate (PrP) was detected in the range of 2.19–112 ng/mL, and butyl p-hydroxybenzoate (BuP) was detected in the range of 0–4.42 ng/mL. TCS was detected in the range of 7.84–460 ng/mL, and TCC was detected in the range of 0–14.2 ng/mL. Hong Wu et al. [18] examined phthalate esters (phthalates), BPs, PBs, BzPs, and TCS in beverage samples collected from the market in South China. The results showed that all of the above endocrine disruptors were detected to varying degrees. Natalia I. Zapata et al. [19] detected TCS in the muscle tissue of fish from rivers in the District of Columbia.

Given the diverse sources and widespread presence of EDCs in food, accurate detection and quantification are crucial for understanding their impact on human health. Up to now, the detection of endocrine disruptors (EDCs) in food has primarily relied on chromatographic methods, particularly gas chromatography (GC) and high-performance liquid chromatography (HPLC) combined with mass spectrometry. Gas chromatography stands out for its ability to effectively separate and quantify EDCs, offering high throughput and sensitivity [20]. It is commonly paired with single-quadrupole or triple-quadrupole mass spectrometry (GC-MS or GC-MS/MS) to enhance detection capabilities. To further improve sensitivity and reduce the risk of false positives, analytes are often derivatized before analysis, which also helps to protect the chromatographic column [21,22]. High-performance liquid chromatography coupled with triple-quadrupole mass spectrometry (HPLC-MS/MS) is a commonly used option for the determination of residues of EDCs due to its high sensitivity and selectivity [23,24]. However, the complexity of food matrices requires appropriate pre-treatment to obtain accurate results. Commonly used pretreatment methods include solid phase extraction (SPE) [25,26,27,28], liquid extraction (LE) [24], dispersive liquid—liquid microextraction (dispersive liquid—liquid microextraction, DLLME) [28], and the QuEChERS method [19,21,23].

Despite the significant health risks posed by EDCs, research on their contamination in honey has been limited. Due to the widespread use of honey in daily life and the simplicity of its processing, researchers have conducted fewer studies on its possible contamination with EDCs in terms of origin, source of honey, packaging materials, and processing. In particular, no previous studies have reported the contamination of EDCs such as TCS, TCC, and MTCS in honey. In addition, in the post-coronavirus pandemic era, which has seen an increase in the use of cleaning products [29], an increase in the consumption of fast food and snacks [30], and a sharp increase in the use of antimicrobials, there is the potential for EDCs to contaminate honey, either through ecological cycling or during processing. These substances, such as TCS and TCC, are widely applied in personal care products and cleaning agents. They can enter the environment through wastewater and eventually contaminate soil and water sources. Bees, while foraging, may come into contact with these pollutants, which can then be transferred to honey. Additionally, the increased use of antimicrobial agents in agriculture to control pests and diseases can lead to the direct contamination of honey through nectar and pollen. This contamination not only affects the quality of honey but also poses potential health risks to consumers due to the presence of endocrine-disrupting chemicals. It is necessary to study the contamination of honey with typical endocrine disruptors and to assess the risk of exposure to typical endocrine disruptors through honey in adults as well as infants. Therefore, it is essential to study the contamination of honey with typical EDCs and to assess the risk of exposure to these chemicals through honey consumption. The purpose of this study is to assess the current situation of TCS, TCC, MTCS, MeP, PrP, BPF, and 4HBP in different packaged honeys from different nectar sources in China and abroad and to assess their associated health risks.

## 2. Experimental Procedure

### 2.1. Reagents and Chemicals

Triclosan (purity ≥ 99%), triclocarban standard (purity ≥ 98%), methyl p-hydroxybenzoate (purity ≥ 98%), and propyl p-hydroxybenzoate (purity ≥ 98%) were purchased from Shanghai Yuanye Biotechnology Co. (Shanghai China). Methyl triclosan standard (purity ≥ 98%) and 4-hydroxybenzophenone (purity ≥ 98%) were purchased from Sigma-Aldrich Co. (St. Louis, MO, USA). Bisphenol F (purity ≥ 98%) was purchased from Beijing Huawei Ruike Chemical Co. (Beijing, China). Methanol (HPLC/ACS grade) was purchased from Beijing Bailing Wei Technology Co. (Beijing, China). N-octanol (AR, purity ≥ 99%), n-butanol (ACS, purity ≥ 99.4%), and n-pentanol (ACS grade) reagents were purchased from Shanghai Aladdin Biochemical Science and Technology Co. (Shanghai China). n-Hexanol (HPLC, purity ≥ 99.5%) was purchased from Beijing Myriad Technology Co. (Beijing, China). Sodium nitrate (purity ≥ 98%) was purchased from Beijing Honghu United Chemical Products Co. (Beijing, China). Ammonium chloride (purity ≥ 99.5%) was purchased from Beijing Tongguang Fine Chemical Company (Beijing, China). Sodium chloride (purity ≥ 99%) and potassium chloride (purity ≥ 99%) were purchased from Sangong Bioengineering Company Limited (Shanghai, China). Aluminum chloride hexahydrate (purity ≥ 99%) was purchased from Beijing Bailing Wei Technology Co. Sodium sulfate (AR) and anhydrous calcium chloride (purity ≥ 96%) purchased from Beijing Huawei Ruike Chemical Co. Sodium hydroxide (purity ≥ 98%) were purchased from Beijing Bailing Wei Technology Co. Ultrapure water is made in the laboratory.

### 2.2. Sample Collection and Preparation

A total of 47 honey samples were purchased from different suppliers, including 40 domestic honeys and 7 foreign (Russia, Spain, France, Cuba, New Zealand, Germany) honeys. This ensures that the results reflect the contamination of endocrine disruptors (EDCs) in honey from different regions, covering 12 different honey sources (acacia, jujube, vitex, multiflora, linden, rape, citrus, loquat, motherwort, sunflower, wolfberry, and milkvetch). This diverse nectar source helps to understand the distribution differences in EDCs in honey from different plant sources. The selected nectar sources cover the common types of honey on the market, ensuring that the results are general and representative. Packaging for honey includes plastic containers (PET, PP), laminated polymer/foil bags, glass bottles with plastic lids, glass bottles with polymer-lined metal lids, and all-glass packages. In total, 15 g of honey was taken and homogenized by stirring for 3 min at 40 °C in a water bath. Subsequently, 10 g of homogenized honey was taken and diluted with 100 mL of ultrapure water to make the sample solution to be tested. Ultra-pure water, which has been purified to a very low level of impurities, provides a relatively pure background for analysis. Therefore, ultra-pure water is used to dilute honey samples.

### 2.3. Instrumentation

In this study, TCS, TCC, MTCS, BPF, 4HBP, MeP, and PrP were detected using high-performance liquid chromatography with a diode array detector (HPLC-DAD). Separation was achieved on a Diamonsil Plus C18 column (250 × 4.6 mm, 5 μm). The column temperature was maintained at 45 °C. The mobile phase consisted of methanol and water. The injection volume was 10 mL, and the flow rate was set at 1.0 mL/min. A binary high-pressure gradient elution program was used: 0–4 min, methanol–water (60/40, *v*/*v*), 4–18 min, methanol–water (80/20, *v*/*v*), 18–23 min, methanol–water (100/0, *v*/*v*), and 23–28 min, methanol–water (60/40, *v*/*v*). In total, 281 nm was used to detect TCS and MTCS, 263 nm to detect TCC, 256 nm to detect MeP and PrP, 277 nm to detect BPF, and 293 nm to detect 4HBP, respectively. The experimental data were processed by LC solution Lite workstation.

### 2.4. DLLME Procedure

The sample to be measured (10 mL, pH 7) was placed in a 15 mL glass centrifuge tube. A solution containing 0.3 g of sodium chloride was added and shaken, and, then, 600 μL of n-octanol was added. After manual shaking, it was sonicated at 20 °C for 5 min to facilitate the extraction process. The sample solution was centrifuged at 3500 rpm for 8 min to achieve phase separation. The upper organic phase was collected using a syringe, and the volume was fixed to 1 mL. After filtration with 0.45 μm organic membrane, HPLC-DAD analysis was performed.

### 2.5. Calculations and Data Processing

The amount of each endocrine disruptor in the honey samples was calculated according to Equation (1).

(1) $$C=\frac{C_{s}*V_{s}}{m_{s}}$$

C (ng/g) is the amount of the target analyte detected in honey. C_S_ (ng/mL) is based on the detected peak area. Concentration of the target analyte in the sample solution was calculated from the standard curve. Vs (mL) is the volume of sample solution. m_S_ (g) is the honey sample mass.

Estimated daily intake (EDI) of each analyte in honey was assessed according to Equation (2).

(2) $$EDI=\frac{C*V}{B}$$

C (ng/g) is the amount of the target analyte detected in honey. V (g/day) is the daily intake of honey. B (kg) is adult weight (50 kg) or infant weight. Infant weight was based on the average weight of infants from the Fifth National Survey of Chinese Children [31].

(3) $$HQ=\frac{EDI}{RfD}$$

(4) $$HED=\frac{NOAEL}{DAF}$$

The health risk assessment in this study utilized the Hazard Quotient (HQ) method, as illustrated in Equation (3) [32]. The reference dose (RfD, ng/kg bw/day) for each analyte is detailed in Table 1. The European Food Safety Authority (2004) [33] recommends an Acceptable Daily Intake (ADI) of 0–107 ng/kg bw/day to limit the total intake of MeP and EtP and their sodium salts. This ADI value was adopted as the RfD for MeP in our study. However, the chronic reference values for TCS, TCC, BPF, 4HBP, and PrP are still being reviewed by regulatory authorities. Consequently, we derived the human RfD from the No Observed Adverse Effect Level (NOAEL) obtained from in vivo experiments. Traditionally, RfD is calculated by dividing the NOAEL by factors accounting for intraspecies and interspecies variability and database uncertainty. The U.S. Environmental Protection Agency further recommends incorporating human equivalence factors (HEDCs) into the oral RfD calculations for these contaminants [34]. To minimize interspecies uncertainty, we extrapolated HED from the NOAEL using 3/4th power allometric scaling of body weight [35].

The HED in Equation (4) was derived from the NOAEL for mice or rats. That is 2.5 × 10^7^ ng/kg bw/day for TCS [36], 2.5 × 10^7^ ng/kg bw/day for TCC [35,36,37], 2 × 10^7^ ng/kg bw/day for BPF [8], 1 × 10^8^ ng/kg bw/day for 4HBP [38], and 6.5 × 10^6^ ng/kg bw/day for PrP [39,40]. The RfD and related parameters were calculated by dividing by the relative dose adjustment factor. Since the RfD value for PrP was derived from immature mice, the RfD value for PrP was additionally adjusted to an uncertainty factor (UF) of 10.

## 3. Results and Discussion

### 3.1. Optimization of DLLME Operation Parameters

The extraction parameters of DLLME include the following: type and amount of extractant, sonication time and sonication temperature, type and amount of inorganic salt, sample pH, and centrifugal speed and centrifugal duration for the optimization of experimental conditions. Maintain the single variable principle during the inquiry process.

#### 3.1.1. Selection of Extractant Type and Dosage

**Figure 1:**
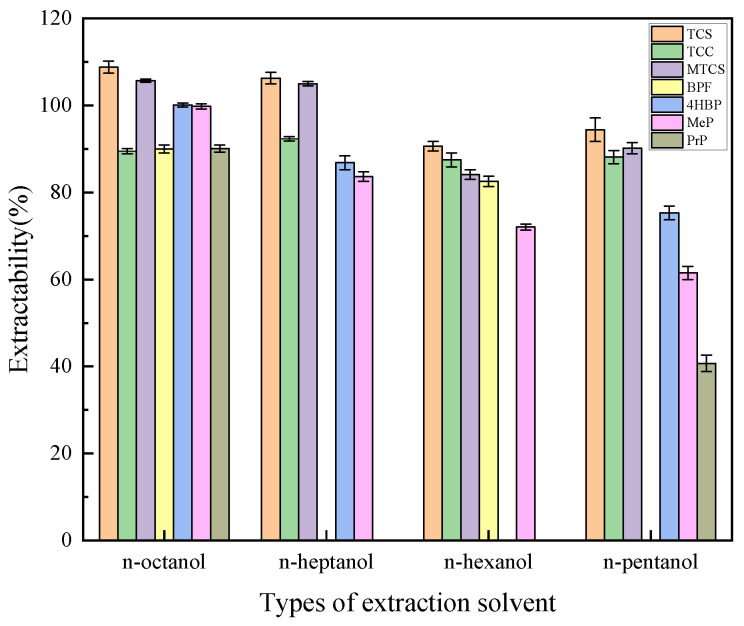
Effects of extractant types on extraction efficiency of 7 typical EDCs.

In total, 600 μL of n-pentanol, n-hexanol, n-heptanol, and n-octanol were selected as extractants to investigate the effect of extractant type on the extraction efficiency of target analytes. As shown in Figure 1, n-octanol showed the highest extraction efficiency for TCS, MTCS, BPF, 4HBP, MeP, and PrP, and n-heptanol showed the highest extraction efficiency for TCC, but the extraction efficiencies for the other target analytes were low. Therefore, n-octanol was finally selected as the extractant.

**Figure 2:**
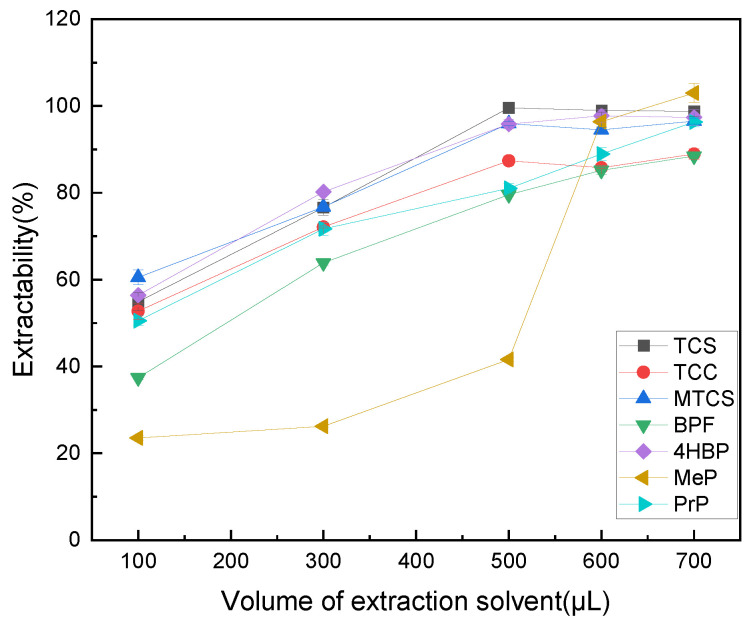
Effect of extractant volume on extraction efficiency of 7 typical EDCs.

The dosage of extractant has a large impact on the experiment, affecting the rate and amount of mass transfer of the target substance, which in turn affects the final extraction efficiency. Therefore, the effect of 100–700 μL of octanol on the extraction efficiency of TCS, TCC, MTCS, BPF, 4HBP, MeP, and PrP was explored. As shown in Figure 2, the extraction efficiencies of TCS, TCC, MTCS, BPS, and 4HBP did not differ much at 600 μL and 800 μL, while the extraction efficiencies of MeP and PrP did not improve much. According to the principle of making each target analyte have a high extraction rate while using as little extractant as possible, 600 μL of n-octanol was finally selected as the optimal extractant dosage.

#### 3.1.2. Selection of Extraction Time and Extraction Temperature

**Figure 3:**
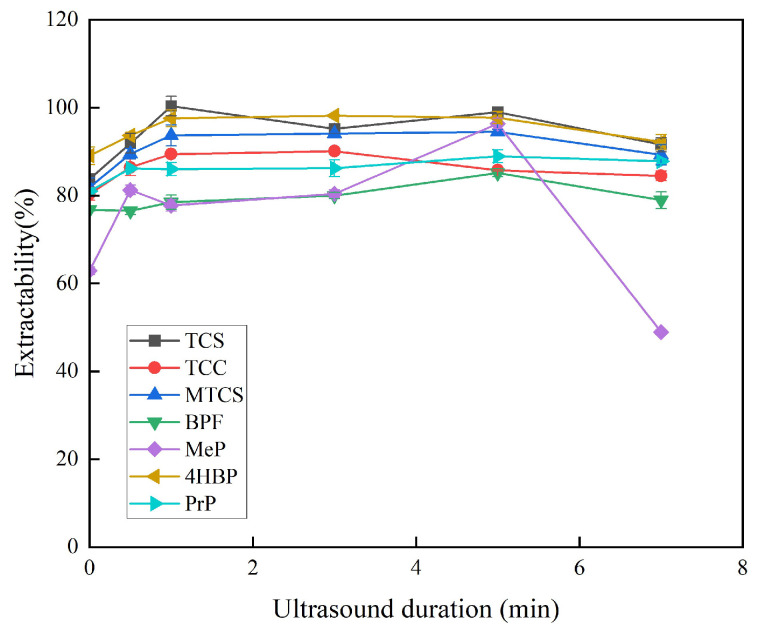
Effect of ultrasonic duration on extraction efficiency of 7 typical EDCs.

In this study, ultrasound was used as a means of dispersion instead of a dispersant, which means that the dispersant solvent can be omitted from the extraction procedure, which is more friendly to the environment. Ultrasound promotes the formation of a fine turbid state during the extraction process, accelerates mass transfer between the two immiscible phases, and reduces equilibrium time, thus contributing to the extraction efficiency. The extraction time in this paper is the sonication time. The sonication time of 0–7 min was chosen for the investigation, and other experimental parameters were kept constant. As shown in Figure 3, the extraction efficiencies of TCS, MTCS, BPF, 4HBP, and PrP increased and then stabilized when the ultrasonication time was 0–5 min. The extraction efficiency of TCC gradually decreased at 3–7 min. The extraction efficiency of MeP reached the maximum at 5 min. The ultrasound duration of 5 min was finally chosen for subsequent experiments.

**Figure 4:**
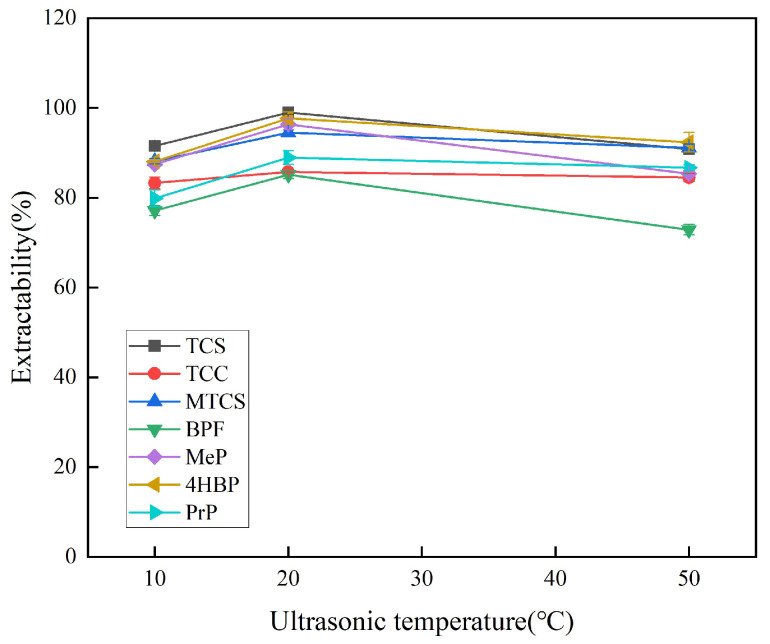
Effect of ultrasonic temperature on extraction efficiency of 7 typical EDCs.

The effect of extraction temperature on the extraction efficiency of target substances at 10 °C, 20 °C, and 50 °C was investigated. As shown in Figure 4, the extraction efficiency is optimal at 20 °C. At lower temperatures, the mass transfer process cannot proceed quickly and adequately, and higher temperatures increase the solubility of the target analyte in water, which in turn leads to lower extraction efficiency. Finally, 20 °C was selected as the optimal sonication temperature.

#### 3.1.3. Selection of Inorganic Salt Type and Dosage

**Figure 5:**
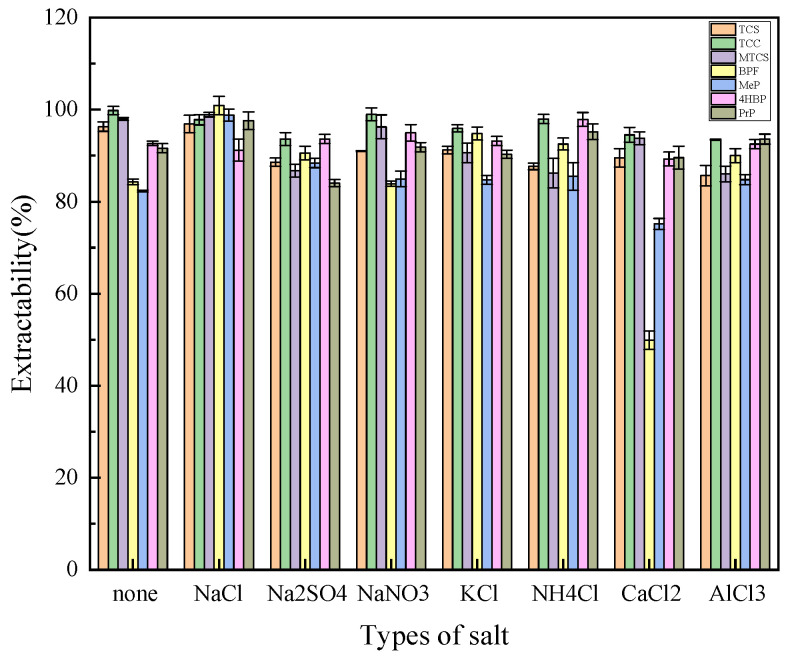
Effects of inorganic salt types on extraction efficiency of 7 typical EDCs.

The salt effect reduces the solubility of the extractant and the target substance in water and improves the extraction efficiency. Na^+^, K^+^, NH_4_^+^, Ca^2+^, and Al^3+^ were selected as the cations to explore, and Cl^−^, SO_4_^2−^, and NO_3_^−^ were selected as the anions to explore based on their valence and species. As shown in Figure 5, the improvement of Na^+^ on the extraction efficiency of MTCS, BPF, MeP, and PrP was more obvious when the amount of salt as well as the anionic species were the same, resulting in a more balanced extraction efficiency of the seven target analytes. So Na^+^ was finally chosen as the best cation. The extraction efficiency of TCC, MTCS, 4HBP, and BPF was significantly improved by Cl^−^ under the condition of salt dosage and cation Na^+^. Therefore, NaCl was finally selected as the best inorganic salt species for the optimization of subsequent experiments.

**Figure 6:**
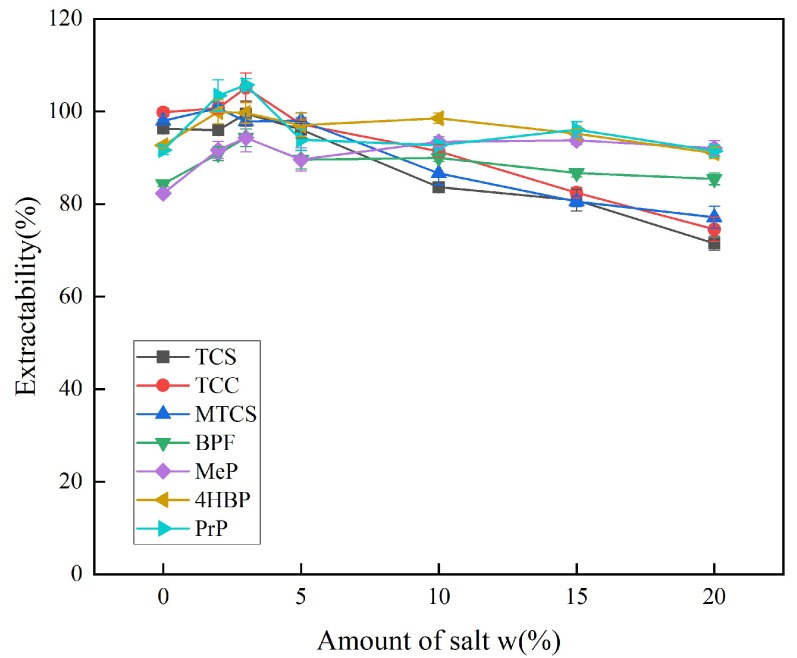
Effect of inorganic salt dosage on extraction efficiency of 7 typical EDCs.

The amount of salt used affects the magnitude of the ionic strength. By appropriately increasing the ionic strength, the salting-out effect can reduce the solubility of the target analyte in water and improve the extraction efficiency. Excessive ionic strength causes an electrostatic effect that prevents the target analyte from entering the extractant, thereby reducing extraction efficiency. Therefore, the effects of different additions of NaCl on the extraction efficiency of the seven targets were compared. As shown in Figure 6, the solubility of the target analyte in the extractant increased when NaCl was added at a mass fraction of 0–3%, which led to an increase in the extraction efficiency. The extraction efficiency of the seven target analytes showed a decreasing trend when the addition of NaCl was increased from 5% to 20% mass fraction. Therefore, a mass fraction of 3% was selected as the optimum amount of NaCl for subsequent experiments.

#### 3.1.4. pH

**Figure 7:**
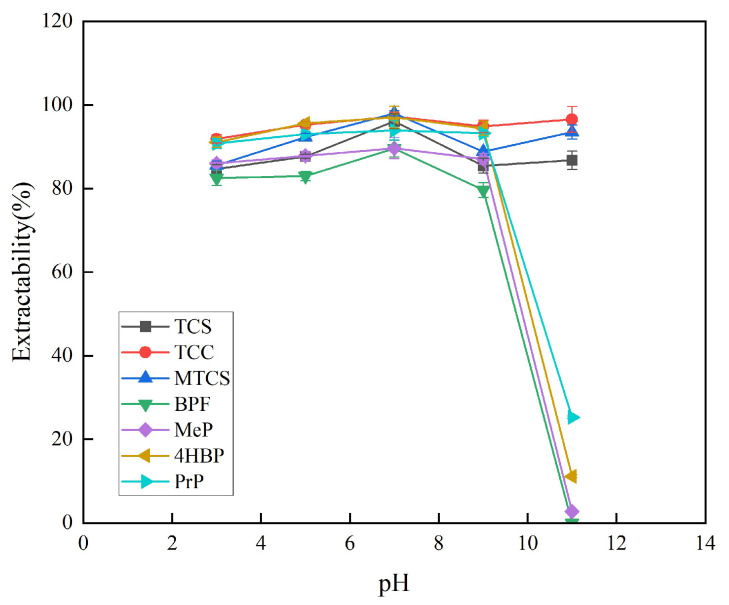
Effect of pH on extraction efficiency of 7 typical EDCs.

The pH of the system was adjusted to 3, 5, 7, 9, and 11 to investigate the influence of different pH levels on the extraction efficiency of the seven target analytes. The experimental results showed that the extraction rate under acidic conditions was overall higher than that under alkaline conditions. The main possible reason for this is that the target analytes under acidic conditions are mainly in the form of un-ionized molecules, which facilitates their partitioning in the organic phase. Under alkaline conditions, the target analyte mainly exists in ionic form, and its water solubility gradually increases, which is not conducive to its dissolution in the organic phase. Under neutral conditions, the target analyte mainly exists in the form of nonionic molecules, which is conducive to its distribution in the organic phase. As shown in Figure 7, under neutral conditions, the extraction efficiency of the seven target analytes is the highest. Finally, pH 7 was chosen for the subsequent experiments.

#### 3.1.5. Selection of Centrifugal Speed and Duration

**Figure 8:**
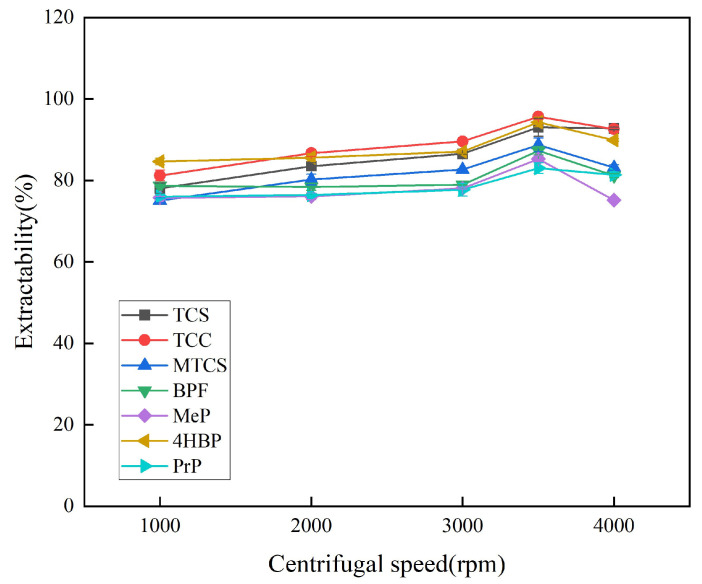
Effect of centrifugal rotation speed on extraction efficiency of 7 typical EDCs.

**Figure 9:**
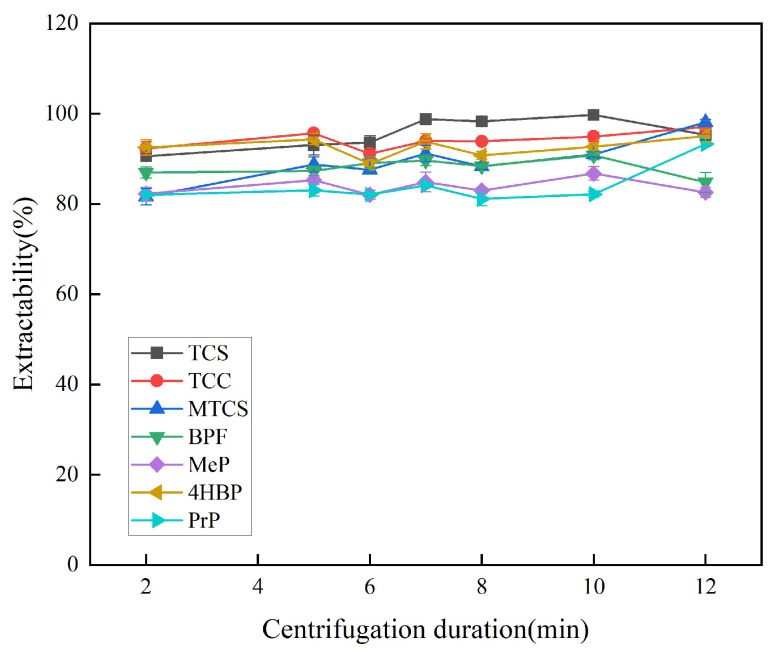
Effect of centrifugation duration on extraction efficiency of 7 typical EDCs.

Centrifugation favors the stratification of the organic and aqueous phases, so the optimal centrifugation speed and duration were explored. At lower rotational speeds, the organic and aqueous phases cannot be completely separated, leading to low extraction efficiency. As shown in Figure 8, the extraction efficiency of the seven targets was optimized at a centrifugal speed of 3500 rpm. Therefore, 3500 rpm was chosen as the optimal centrifugal speed, and the optimal centrifugal duration was explored on this basis. As shown in Figure 9, the extraction efficiency of the target was enhanced when the centrifugation duration was increased from 2 min to 7 min. The overall extraction efficiencies of TCS, TCC, MTCS, BPF, 4HBP, and PrP were high and stable at 8 min. In order to ensure the stability of the experimental results, 8 min was finally selected as the optimal centrifugation duration.

### 3.2. Method Validation

The proposed method was verified in terms of sensitivity, linearity, accuracy, and precision. In order to ensure the accuracy and precision of honey sample detection, each compound in the sample was spiked at low, medium, and high levels. The low-level spiked concentrations of TCS, TCC, MTCS, BPF, 4HBP, MeP, and PrP were 20, 5, 50, 20, 5, 5, and 5 μg/L, respectively. The medium-level spiked concentrations of TCS, TCC, MTCS, BPF, 4HBP, MeP, and PrP were 60, 20, 150, 100, 30, 30, and 30 μg/L, respectively. The high-level spiked concentrations of TCS, TCC, MTCS, BPF, 4HBP, MeP, and PrP were 150, 40, 300, 200, 60, 100, and 60 μg/L, respectively.

A UALLME-HPLC-DAD method for the simultaneous determination of TCS, TCC, MTCS, BPF, 4HBP, MeP, and PrP in honey was finally developed and evaluated for sensitivity, linearity, accuracy, and precision. The accuracy of the optimized method was evaluated by recovery experiments, as shown in Table 2, and the actual sample spiking recoveries were in the range of 89.70–102.2%, RSD values were in the range of 1.1–3.9%, intra-day precision was in the range of 0.6–1.6%, and inter-day precision was in the range of 0.2–1.2%.

### 3.3. Real Sample Analysis

#### 3.3.1. Analysis of the Distribution of EDCs in Honey from Different Nectar Sources

The honey types tested in this study included seven types of honey for which relevant databases have been established: acacia honey, jujube honey, vitex honey, linden honey, rape flower honey, citrus honey, and sunflower honey, as well as multifloral honey, loquat honey, motherwort honey, wolfberry honey, and milk vetch honey, which are commonly found on the market, for a total of 12 types of honey sources and 47 samples. In this experiment, a total of 11 kinds of honey from different sources were detected and analyzed, including 9 acacia honey samples, 4 jujube honey samples, 4 jujube honey samples, 3 vitex flower samples, 3 sunflower rice samples, 3 citrus honey samples, 3 rape flower samples, 13 multifloral honey samples, 1 loquat honey sample, 1 sample of motherwort honey, and 1 sample of milk vetch honey (shown in Tables S1–S5).

TCS, TCC, BPF, 4HBP, MeP, and PrP were detected in honey samples at different concentrations, and MTCS was not detected in all honey samples. TCS was detected in 14 honey samples, and TCC was detected in 9 honey samples. As shown in Table 3, TCS was detected in honey samples from acacia, jujube, linden, rape, citrus, multifloral, and wolfberry sources, with a total detection rate of 29.79%. The greatest frequency of detection of TCS was found in citrus honey, with a frequency of 66.67%. The maximum concentration of TCS was found in linden honey at 144.6 μg/kg.

As shown in Table 4, TCC was detected in honey samples from the sources of acacia, jujube, multiflora, sunflower, motherwort, and wolfberry, with a total detection rate of 19.15%. TCC was detected with the greatest frequency in the motherwort honey, with a frequency of 100%, but was detected at concentrations lower than the LOQ.

BPF had the highest detection rate among the seven typical endocrine disruptors. As shown in Table 5, BPF was detected in honey from all honey types with a total detection rate of 97.87%. It was detected in 88.89% of acacia honey samples and 100% of honey samples from other honey sources. The maximum detectable concentration was found in multifloral honey with a concentration of 1193 μg/kg.

As shown in Table 1, 4HBP was not detected in honey samples from the sources of linden, loquat, motherwort and zoysia japonica, with a total detection rate of 36.17%. 4HBP was detected most frequently in multifloral honey with a concentration of 69.23%, and the maximum detection concentration occurred in multifloral honey at 294.9 μg/kg.

The results showed that MeP and PrP were detected in honey samples from 12 honey sources, despite the fact that the Chinese national standard GB2760-2011 stipulates that the addition of propylparaben and its sodium salt is not allowed in food. As shown in Table 6, the total detection rate of MeP was 82.98%. The greatest frequency of detection was found in acacia honey, bramble honey, rape honey, citrus honey, loquat honey, motherwort honey, wolfberry honey, and milk vetch honey, all with 100% detection concentration. The maximum detected concentration was 439.5 μg/kg in milk vetch honey. The total detection rate of PrP in honey samples was 80.85%. The highest detection frequency of 100% was found in acacia honey, citrus honey, loquat honey, sunflower honey, motherwort honey, wolfberry honey, and milk vetch honey. The maximum detected concentration was found in multifloral honey, which was 136.7 μg/kg.

**Figure 10:**
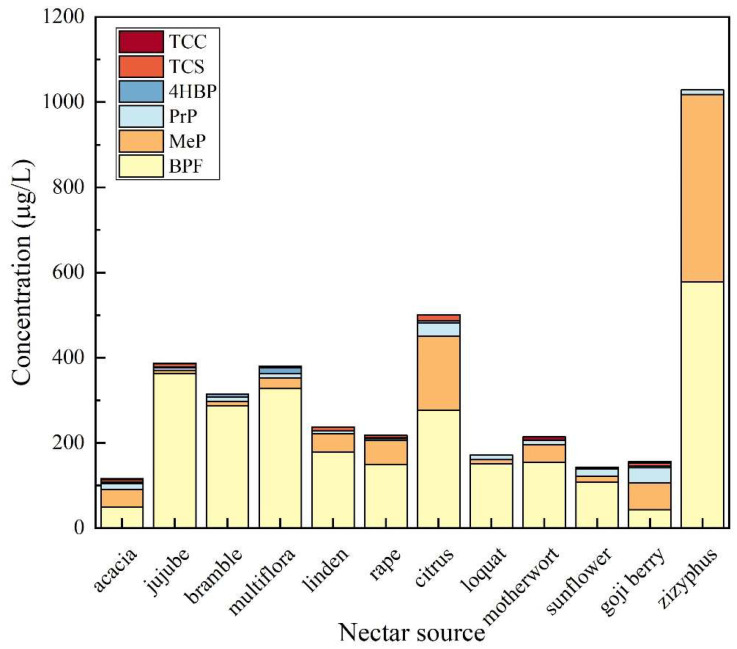
Contamination of TCS, TCC, BPF, 4HBP, MeP, and PrP in honey from different sources.

As shown in Figure 10, the experimental results indicated that the total detection rate of TCS was 29.79% in the above 12 honey sources. The highest detection frequency of TCS was 66.67% in citrus honey, followed by date honey (50%), linden honey (50%), and wolfberry honey (50%). TCS was the most contaminated in linden honey with a concentration of 144.6 μg/kg, followed by rape honey with a concentration of 121 μg/kg. The total detection rate of TCC in honey was 19.15%. The highest detection frequency of TCC was found in motherwort honey with 100%, followed by wolfberry honey (50%). The overall contamination of TCC in honey was low, with all detected concentrations lower than the LOQ. In the honey samples, BPF was detected with high frequency and high levels of contamination, with a total detection frequency of 97.87%. The detection rate of BPF was 100% in jujube honey, vitex honey, linden honey, rape honey, citrus honey, loquat honey, multifloral honey, sunflower honey, motherwort honey, wolfberry honey, and milk vetch honey, and the lowest detection rate was 88.89% in acacia honey. The maximum detected concentration of BPF occurred in multifloral honey, which was 1194 μg/kg; thus, the significance of EDCs needs to be emphasized. The total detection rate of 4HBP in honey was 36.17%. 4HBP was detected most frequently in multifloral honey with 69.23%, followed by wolfberry honey (50%). The highest concentration of 4HBP was detected in multifloral honey, with a detection concentration of 294.9 μg/kg, indicating a wide range and high degree of 4HBP contamination in multifloral honey. MeP and PrP were detected in all honey sources with high frequency, indicating a wide range and high degree of contamination of MeP and PrP in honey. The total detection rate of MeP was 83%, and the detection frequency of MeP in acacia honey, bramble honey, rape honey, citrus honey, loquat honey, motherwort honey, wolfberry honey, and milk vetch honey was 100%, followed by linden honey (75%). MeP was detected at the maximum concentration of 439.5 μg/kg in milk vetch honey. The total detection rate of PrP was 87%. PrP was detected at 100% in acacia honey, citrus honey, loquat honey, sunflower honey, motherwort honey, wolfberry honey, and milk vetch honey, followed by jujube honey (75%) and linden honey (75%). Among them, PrP was detected in multifloral honey at the highest concentration of 136.7 μg/kg.

MTCS was not detected in any of the honeys from the above 12 honey sources. The largest numbers of typical endocrine disruptors were detected in acacia honey, jujube honey, multifloral honey, and wolfberry honey, with a total of six typical endocrine disruptors detected, including TCS, TCC, BPF, 4HBP, MeP, and PrP. A total of five typical endocrine disruptors including TCS, BPF, 4HBP, MeP, and PrP were detected in rape and citrus honey. Five typical endocrine disruptors including TCC, BPF, 4HBP, MeP, and PrP were detected in sunflower honey. Four typical endocrine disruptors including BPF, 4HBP, MeP, and PrP were detected in vitex honey. Three typical endocrine disruptors including TCC, BPF, 4HBP, MeP, and PrP were detected in vitex honey. Three typical endocrine disruptors including TCC, 4HBP, MeP, and PrP were detected in sunflower honey. Three typical endocrine disruptors including TCC, 4HBP, MeP, and PrP were detected in vitex honey. Four typical endocrine disruptors including TCS, BPF, MeP, and PrP were detected in linden honey. Four typical endocrine disruptors including TCC, BPF, MeP, and PrP were detected in motherwort honey. Three typical endocrine disruptors including BPF, MeP, and PrP were detected in loquat honey and zi yun ying honey. The maximum detected concentrations of BPF, 4HBP, and PrP were from multifloral honey, with concentrations of 1193 μg/kg, 294.9 μg/kg, and 136.7 μg/kg, respectively. The maximum detected concentration of TCS was from citrus honey, with a concentration of 144.6 μg/kg. The maximum detected concentration of MeP was derived from the honey of milk vetch with a concentration of 439.5 μg/kg.

#### 3.3.2. Analysis of Honey Contamination by EDCs in Packaging Made of Different Materials

There were five types of honey packaging involved in this study, including plastic containers (PET, PP), laminated polymer/foil pouches, glass bottles with plastic lids, glass bottles with polymer-lined metal lids, and all-glass bottle packaging. In this study, seven typical endocrine disruptors were detected in honey samples with different packaging materials. In this experiment, a total of 31 honey samples were tested in plastic containers, including 28 PET packages and 3 PP packages. Five samples of honey in laminated polymer/aluminum foil packaging, seven samples of honey in glass bottles with plastic lids, three samples of honey in glass bottles with polymer-lined metal lids, and one sample of honey in all-glass packaging were tested, as shown in Tables S6–S8. The results showed that TCC, BPF, 4HBP, MeP, and PrP were detected in honey samples from all five packaging materials.

For the triclosan group, as shown in Table 7, the total detection rate of TCS in honey samples from the five packaging materials was 29.79%. Among them, TCS was not detected in honey samples packaged in laminated polymer/foil pouches; the highest detection rate was found in the packaging of glass bottles with plastic lids with a detection frequency of 42.86%, and the highest concentration of TCS was detected in PET packaging with a concentration of 144.6 μg/kg. The total detection of TCC in honey samples of five different packaging materials was 19.15%. The highest detection rate of 33.33% was found in honey samples packaged in PP and glass bottles with polymer-lined metal lids. TCC was detected at lower concentrations than the LOQ in honey samples of different packaging materials.

The total detection rate of BPF in honey samples of five different packaging materials was 97.87%. As shown in Table 8, BPF was detected in 96.43% of the honey samples packed in PET and 100% of the honey samples packed in all other packaging materials, and the maximum concentration of BPF was detected in the honey samples packed in PET, which was 1193 μg/kg.

The total detection rate of 4HBP in honey samples from the five packaging materials was 36.17%. As shown in Table 9, 4HBP had the highest detection rate of 57.14% in honey samples packaged in glass bottles with plastic lids and the highest concentration of 294.9 μg/kg in honey samples packaged in laminated polymer/foil bags.

For parabens, MeP was detected in 82.98% of honey samples in all five packaging materials, and PrP was detected in 80.85% of honey samples in all five packaging materials. As shown in Table 10, MeP had the highest detection rate of 100% in honey samples packed in PP packaging, glass bottles with polymer-lined metal lids, and all-glass packaging. The concentration of MeP was the highest in the honey sample packed in PET, with a detection concentration of 439.5 μg/kg. For PrP, the maximum detection was found in honey samples packaged in glass bottles with polymer-lined metal lids at 100%, and the highest detected concentration was found in honey samples packaged in PET at 136.7 μg/kg.

**Figure 11:**
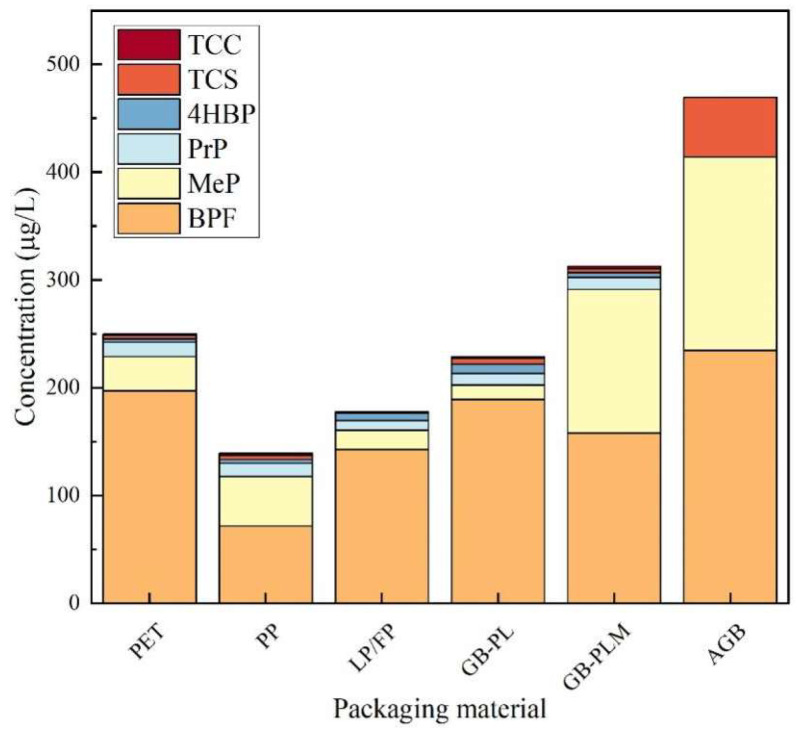
Contamination of TCS, TCC, BPF, 4HBP, MeP, and PrP in honey of different packaging materials; LP/FP: laminated polymer/foil bags; GB-PL: glass bottles with plastic lids; GB-PLM: glass bottles with polymer-lined metal lids; and AGB: all-glass bottle.

As shown in Figure 11, the experimental results indicated that the highest detected concentrations of TCS, BPF, MeP, and PrP were found in PET-packed honey, with the detected concentrations of 144.6 μg/kg, 1193 μg/kg, 439.5 μg/kg, and 136.7 μg/kg, respectively, indicating that TCS, BPF, MeP, and PrP were more seriously contaminated in PET-packed honey. The maximum detection frequencies of TCS and 4HBP were found in honey packed in glass bottles with plastic lids, with detection frequencies of 42.86% and 57.14%, respectively, indicating that the contamination range of TCS and 4HBP in honey packed in glass bottles with plastic lids was wide. The maximum detection frequency of TCC was found in honey packed in PP and glass bottles with polymer-lined metal lids, with detection frequencies of 33.33%, indicating a wide range of TCC contamination in the above two types of packaged honey. The highest detection frequency of MeP was found in honey packaged in PP, glass bottles with polymer-lined metal lids, and all-glass packages, all with 100% detection rates, indicating a wide range of MeP contamination in these three types of packaged honey. BPF was detected in 96.43% of the honey packed in PET and 100% of the honey packed in the other four materials. This indicates that BPF has a wide range of contamination in honey packaged in all materials. The highest detection frequency of PrP was found in honey packaged in glass bottles with polymer-lined metal lids, with a detection frequency of 100%, which indicates that the range of contamination of PrP is wide in this package.

TCS and TCC were detected in honey samples; although the number of samples detected was small (29.79% for TCS and 19.15% for TCC) and the concentrations were low (the maximum detected concentration was 144.6 μg/kg for TCS, and the concentrations for TCC were below the LOQs), they still indicate that TCS and TCC contaminated the honey. BPF (97.87%), MeP (82.98%), and PrP (80.85%) were detected in almost all honey samples, and 4HBP (36.17%) was detected in a larger number of samples. This reflects the widespread use of benzophenones, parabens, and bisphenols. For benzophenones, Japan and Italy stipulate that the maximum amount should not exceed 0.3% when used in contact with food products. For BPF, China and Korea stipulate that its specific migration level in food contact materials should not be higher than 50 μg/kg, but, in this study, BPF was detected in concentrations up to 1193 μg/kg. For parabens, China’s National standard GB2760-2014 stipulates that MEP, ETP, and the total amount of sodium salts in various foods range from 12 ng/g (fresh fruits and vegetables) to 500 ng/g (filling materials and dough in baking foods). However, despite reports of the reproductive toxicity of PrP in animal and in vitro studies, PrP has not yet been included in the regulatory list for food additives. Additionally, the Chinese national standard GB2760-2011 states that applications for production licenses for PrP and its sodium salt as food additives are not being entertained.

#### 3.3.3. Comparison of Contamination of Honey at Home and Abroad

In this experiment, honey samples from different regions of China were tested. It covers seven regions of China: North, Northeast, East, Central, South, Northwest, and Southwest China. In addition, honey samples from abroad were also tested, including the following: Russia, Spain, France, Cuba, New Zealand, and Germany. Tables S9 and S10 show the detection of seven typical endocrine disruptors in honey samples at home and abroad.

As shown in Table 11, BPF, PrP, MeP, 4HBP, TCS, and TCC were detected in 97.5%, 85%, 82.5%, 32.5%, 27.5%, and 20% of honey samples originating from China, respectively, and MTCS was not detected. In honey samples of foreign origin, BPF, MeP, 4HBP, PrP, TCS, and TCC were detected at frequencies of 100%, 85.71%, 57.17%, 57.14%, 42.86%, and 14.28%, respectively, and MTCS was not detected. The highest concentrations of TCS, 4HBP, and MeP in honey samples were found in samples from China, with concentrations of 144.6 μg/kg, 294.9 μg/kg, and 439.5 μg/kg, respectively. The highest concentrations of BPF and PrP in honey samples were found in foreign samples, with concentrations of 1193 μg/kg and 136.7 μg/kg. The maximum detected concentration of TCC in both domestic and foreign honey samples was lower than that of LOQ. This shows that, for TCS, BPF, 4HBP, and MeP, the contamination range of foreign samples is wider than that of domestic samples, and, for TCC and PrP, the contamination range of domestic samples is wider.

**Figure 12:**
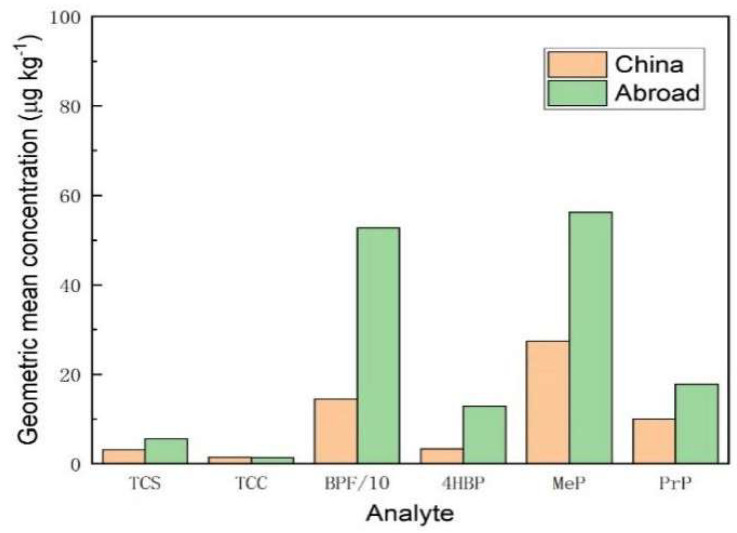
Comparative analysis of geometric mean concentrations of 6 typical EDCs in honey samples from home and abroad.

As shown in Figure 12, for the geometric mean concentrations of TCS, BPF, 4HBP, MeP, and PrP, foreign honey samples were higher than Chinese honey samples. The geometric mean concentrations of TCC were comparable in both domestic and foreign samples. It can be seen that the contamination of typical endocrine disruptors in foreign honey samples was more serious than domestic ones. Among them, BPF had the highest level of contamination with a geometric mean concentration of 527.1 μg/kg, followed by MeP with a geometric mean concentration of 56.19 μg/kg.

### 3.4. Daily Intake and Health Risk Assessment

#### Daily Intake and Health Risk Assessment for Adults

The adult EDI and RfD for TCS, TCC, BPF, 4HBP, MeP, and PrP are shown in Table 12. The EDI was calculated as the maximum detected concentration of EDCs in the honey samples, and the LOD was used for detected concentrations below the LOQ. The highest EDIs for a single intake of TCS, TCC, BPF, 4HBP, MeP, and PrP were 28.92, 1.60, 238.6, 58.98, 87.90, and 27.34 ng/kg bw/d, respectively, which were all lower than the calculated RfD, when calculated for a 50 kg adult consuming 10 g of honey per day. It suggests that none of the six typical endocrine disruptors mentioned above ingested through honey in adults pose a significant risk to human health if 10 g of honey is consumed daily. The lower thresholds were chosen for this study and adjusted for possible uncertainties (Table 12). Considering that there are a number of uncertainties in the risk assessment, including the RfD, which found that TCS produces a more severe health damage endpoint (i.e., liver injury) [36]. RfD levels are lower on the more sensitive endpoint of hormone reduction due to intraspecific and interspecific uncertainty [36].

The HQ has been used to evaluate the non-carcinogenic health risk of a pollutant and has a specific threshold value. Significant health risks exist when the HQ > 1. This study incorporated the human equivalent dose into the risk assessment (Table 12). The HQ of TCS, TCC, BPF, 4HBP, MeP, and PrP ingestion from honey in adults were 2.2 × 10^−3^, 1.2 × 10^−4^, 2.2 × 10^−2^, 1.1 × 10^−3^, 8.79 × 10^−6^, and 0.15, respectively. Although the HQ of all six EDCs was less than 1, with PrP having the highest HQ of 0.15, it still needs to be a cause for concern. PrP is a paraben frequently used in cosmetics and food and has been approved for use in several over-the-counter drugs [11,41]. However, PrP is more toxic than MeP, causing stress and inflammation, damaging DNA and fatty acid metabolism, and its low estrogen and anti-androgen effects can interfere with normal reproductive function [42]. It is reported that PrP has reproductive toxicity to both male and female rats [43].

**Table 12:** Adult daily intakes and reference measures for TCS, TCC, BPF, 4HBP, MeP, and PrP.

EDCs	Maximum Detectable Concentration (μg/kg)	RfD (ng/kg bw/d)	EDI (ng/kg bw/d)	HQ	Reference Measurements ^a^ and Uncertainty Factors
TCS	144.6	1.3 × 10^4^	28.92	2.2 × 10^−3^	HED derived from mature rats: 4.0 × 10^6^ ng/kg bw/day Uncertainty factor for infants: 3 (inter-) × 10 (intraspecies) × 10 (DBU ^b^)
TCC	8	1.3 × 10^4^	1.60	1.2 × 10^−4^	HED derived from mature rats: 4.0 × 10^6^ ng/kg bw/day Uncertainty factor for infants: 3 (inter-) × 10 (intraspecies) × 10 (DBU ^b^)
BPF	1193	1.1 × 10^4^	238.6	2.2 × 10^−2^	HED derived from mature rats: 3.2 × 10^6^ ng/kg bw/day Uncertainty factor for infants: 3 (inter-) × 10 (intraspecies) × 10 (DBU ^b^)
4HBP	294.9	5.3 × 10^4^	58.98	1.1 × 10^−3^	HED derived from mature rats: 1.6 × 10^7^ ng/kg bw/day Uncertainty factor for infants: 3 (inter-) × 10 (intraspecies) × 10 (DBU ^b^)
MeP	439.5	1.0 × 10^7^	87.90	8.79 × 10^−6^	EDI: 1.0 × 10^7^ ng/kg bw/day for total MeP and EtP
PrP	136.7	1.77 × 10^2^	27.34	0.15	HED derived from immature mice: 5.3 × 10^5^ ng/kg bw/day Uncertainty factor for infants: 3 (inter-) × 10 (intraspecies) × 10 (DBU ^b^) × 10 (for adults)

^a^: RfD: reference dose, EDI: each daily intake, and HED: human equivalent dose. The EDI of the total MeP and EtP is proposed by the European Food Safety Authority (2004) [33]. The HED values of TCS [36], TCC [37], BPF [8], 4HBP [38], and PrP [40,44,45] are derived from NOAELs (2.5 × 10^7^, 2.5 × 10^7^, 2 × 10^7^, 1 × 10^8^, and 6.5 × 10^6^, ng/kg bw/day, respectively) observed in CD-1 mice or Sprague Dawley rats by multiplying with relative dosimetric adjustment factors (6.2, 6.2, 6.2, 6.2, and 12.3, respectively) [35]; ^b^: Database uncertainty accounts for the lack of the multigenerational reproductive studies, the lack of adequate developmental studies, and the lack of adequate repeat-dose studies in at least two mammalian species.

## 4. Conclusions

In this study, a method for the simultaneous determination of seven typical endocrine disruptors (TCS, TCC, MTCS, BPF, 4HBP, MeP, and PrP) in honey by ultrasound-assisted dispersive liquid–liquid microextraction coupled with HPLC was developed. Actual samples were also tested on a total of 47 honey samples from 7 countries, 5 different packaging materials, and 12 honey sources. The results showed that six EDCs, including TCS, TCC, BPF, 4HBP, MeP, and PrP, were detected in honey samples except MTCS. BPF, MeP, and PrP were detected in all honey sources. The total detection rates of 4HBP, TCS, and TCC in honey samples were 36.17%, 29.79%, and 19.15%, respectively. The maximum detected concentration of BPF was 1194 μg/kg, which originated from multifloral honey packed in PET in foreign countries. The maximum detected concentration of MeP was 439.5 μg/kg, which originated from milk vetch honey packed in PET in China. The maximum detected concentration of 4HBP was 294.9 μg/kg, which originated from domestic multifloral honey packaged in laminated polymer/foil pouches. The maximum detected concentration of TCS was 144.6 μg/kg, which originated from domestic linden honey packaged in PET. The maximum detected concentration of PrP was 136.7 μg/kg, which originated from foreign multifloral honey packaged in PET. TCC was lower than LOQ in all honey samples. A total of 136.7 μg/kg was derived from polyfloral honey packaged in foreign PET. TCC was detected at lower concentrations than LOQ in all honey samples. The contamination of TCS, BPF, 4HBP, and MeP in foreign honey samples was wider than domestic. The contamination of TCS, 4HBP, and MeP in domestic honey samples was more extensive than that in foreign countries, and the contamination of BPF and PrP in foreign honey samples was more extensive than that in domestic countries. Health risk assessments indicated low risks for adults but high risks for children under one year old, particularly from BPF exposure. We recommend that children avoid honey and that the contamination monitoring of endocrine disruptors in food processing be enhanced. The test results of honey samples show that endocrine disruptors such as BPF are widely present. The pollution sources of these typical endocrine disruptors still need to be analyzed in depth, so as to propose effective measures to control the quality of honey and improve the pollution situation of typical endocrine disruptors in honey. Furthermore, the method established in this paper is currently only applied to honey matrix samples. The application of typical endocrine disruptors in other food matrices also awaits further research.

## Figures and Tables

**Table 1:** Detection of 4HBP in honey from different sources.

	Nectar Source	Detection Rate (%)	Range (μg/kg)
4HBP	acacia honey	22.22 (2/9)	ND ^a^-50.62
	jujube honey	25 (1/4)	ND- < LOQ ^b^
	vitex honey	33.33 (1/3)	ND-172.3
	linden flower	0 (0/4)	ND
	rape flower honey	33.33 (1/3)	ND- < LOQ
	citrus honey	33.33 (1/3)	ND-94.43
	loquat honey	0 (0/1)	ND
	multifloral honey	69.23 (9/13)	ND-294.9
	sunflower honey	33.33 (1/3)	ND- < LOQ
	motherwort honey	0 (0/1)	ND
	wolfberry honey	50 (1/2)	ND- < LOQ
	milk vetch honey	0 (0/1)	ND

^a^: No detection; ^b^: Below the LOQ.

**Table 2:** Precision of method for detection of 7 typical EDCs by UALLME-HPLC-DAD.

Analyte		TCS	TCC	MTCS	BPF	4HBP	MeP	PrP
Linear range (μg L^−1^)		200–1500	25–500	500–3000	200–3000	50–1000	10–2000	50–1000
Correlation coefficient R^2^		0.9995	0.9996	0.9991	0.9996	0.9994	0.9999	0.9994
Limit of detection (μg L^−1^)		55	8	127	43	15	10	11
Limit of quantification (μg L^−1^)		184	25	422	143	50	36	38
Standard recovery(%)	Low spiked level	96.82	98.90	90.02	89.70	99.52	98.27	98.99
	Mean spiked level	100.4	98.31	97.59	95.81	95.77	94.44	100.7
	High spiked level	100.2	102.2	95.98	98.48	100.0	97.54	94.11
Relative standard deviation (*n* = 9) (%)		1.7–2.2	1.3–2.8	1.1–2.7	1.4–3.2	1.8–3.9	1.3–3.4	1.2–2.4
Inter-day variability (n = 6) (%)		0.8	1.5	0.6	1.1	1.1	0.9	1.6
Intra-day variability (n = 6) (%)		1.2	0.5	0.5	0.9	0.3	0.2	0.3

**Table 3:** Detection of TCS in honey from different sources.

	Nectar Source	Detection Rate (%)	Range (μg/kg)
TCS	acacia honey	44.44	ND ^a^- < LOQ ^b^
	jujube honey	50	ND- < LOQ
	vitex honey	0	ND
	linden flower	50	ND-144.6
	rape flower honey	33.33	ND-121
	citrus honey	66.67	ND- < LOQ
	loquat honey	0	ND
	multifloral honey	15.38	ND- < LOQ
	sunflower honey	0	ND
	motherwort honey	0	ND
	wolfberry honey	50	ND- < LOQ
	milk vetch honey	0	ND

^a^: No detection; ^b^: Below the LOQ.

**Table 4:** Detection of TCC in honey from different sources.

	Nectar Source	Detection Rate (%)	Range (μg/kg)
TCC	acacia honey	33.33 (3/9)	ND ^a^- < LOQ ^b^
	jujube honey	25 (1/4)	ND- < LOQ
	vitex honey	0 (0/3)	ND
	linden flower	0 (0/4)	ND
	rape flower honey	0 (0/3)	ND
	citrus honey	0 (0/3)	ND
	loquat honey	0 (0/1)	ND
	multifloral honey	15.38 (2/13)	ND- < LOQ
	sunflower honey	33.3 3(1/3)	ND- < LOQ
	motherwort honey	100 (1/1)	<LOQ
	wolfberry honey	50 (1/2)	ND- < LOQ
	milk vetch honey	0 (0/1)	ND

^a^: No detection; ^b^: Below the LOQ.

**Table 5:** Detection of BPF in honey from different sources.

	Nectar Source	Detection Rate (%)	Range (μg/kg)
BPF	acacia honey	88.89 (8/9)	ND ^a^-612.5
	jujube honey	100 (4/4)	232.1–642.4
	vitex honey	100 (3/3)	224.7–415.2
	linden flower	100 (4/4)	<LOQ ^b^-593.7
	rape flower honey	100 (3/3)	<LOQ-297.9
	citrus honey	100 (3/3)	190.7–376.7
	loquat honey	100 (1/1)	150.8
	multifloral honey	100 (13/13)	<LOQ-1193
	sunflower honey	100 (3/3)	<LOQ-189
	motherwort honey	100 (1/1)	154.6
	wolfberry honey	100 (2/2)	<LOQ
	milk vetch honey	100 (1/1)	578.2

^a^: No detection; ^b^: Below the LOQ.

**Table 6:** Detection of MeP and PrP in honey from different sources.

	Nectar Source	Detection Rate (%)	Range (μg/kg)
MeP	acacia honey	100 (9/9)	<LOQ ^b^-176.9
	jujube honey	50 (2/4)	ND ^a^-64.86
	vitex honey	100 (3/3)	<LOQ
	linden flower	75 (3/4)	ND-249.7
	rape flower honey	100 (3/3)	<LOQ-149.5
	citrus honey	100 (3/3)	89.65–299.2
	loquat honey	100 (1/1)	<LOQ
	multifloral honey	69.23 (9/13)	ND-320.9
	sunflower honey	66.67 (2/3)	ND-70.02
	motherwort honey	100 (1/1)	40.52
	wolfberry honey	100 (2/2)	54.34–72.19
	milk vetch honey	100 (1/1)	439.5
PrP	acacia honey	100 (9/9)	<LOQ-109.3
	jujube honey	75 (3/4)	ND- < LOQ
	vitex honey	33.33 (1/3)	<LOQ
	linden flower	75 (3/4)	ND- < LOQ
	rape flower honey	66.67 (2/3)	ND- < LOQ
	citrus honey	100 (3/3)	<LOQ-56.86
	loquat honey	100 (1/1)	<LOQ
	multifloral honey	69.23 (9/13)	ND-136.7
	sunflower honey	100 (3/3)	<LOQ-39.42
	motherwort honey	100 (1/1)	<LOQ
	wolfberry honey	100 (2/2)	<LOQ-120.3
	milk vetch honey	100 (1/1)	<LOQ

^a^: No detection; ^b^: Below the LOQ.

**Table 7:** Detection of TCS and TCC in honey with different packaging materials.

	Packaging Material	Detection Rate (%)	Range (μg/kg)
TCS	PET	28.57 (8/28)	ND ^a^-144.6
	PP	33.33 (1/3)	ND- < LOQ ^b^
	laminated polymer/foil pouches	0 (0/5)	ND
	glass bottles with plastic lids	42.86 (3/7)	ND- < LOQ
	glass bottles with polymer-lined metal lids	33.33 (1/3)	ND- < LOQ
	all-glass bottle	100 (1/1)	<LOQ
TCC	PET	17.86 (5/28)	ND- < LOQ
	PP	33.33 (1/3)	ND- < LOQ
	laminated polymer/foil pouches	20 (1/5)	ND- < LOQ
	glass bottles with plastic lids	14.28 (1/7)	ND- < LOQ
	glass bottles with polymer-lined metal lids	33.33 (1/3)	ND- < LOQ
	all-glass bottle	0 (0/1)	ND

^a^: No detection; ^b^: Below the LOQ.

**Table 8:** Detection of BPF in honey with different packaging materials.

	Packaging Material	Detection Rate (%)	Range (μg/kg)
BPF	PET	96.43 (27/28)	ND ^a^-1193
	PP	100 (3/3)	<LOQ ^b^-199.3
	laminated polymer/foil pouches	100 (5/5)	<LOQ-580.4
	glass bottles with plastic lids	100 (7/7)	ND-612.5
	glass bottles with polymer-lined metal lids	100 (3/3)	<LOQ-479.8
	all-glass bottle	100 (1/1)	234.5

^a^: No detection; ^b^: Below the LOQ.

**Table 9:** Detection of 4HBP in honey with different packaging materials.

	Packaging Material	Detection Rate (%)	Range (μg/kg)
4HBP	PET	32.14 (9/28)	ND ^a^-172.3
	PP	33.33 (1/3)	ND-50.62
	laminated polymer/foil pouches	40 (2/5)	ND-294.9
	glass bottles with plastic lids	57.14 (4/7)	ND-72.37
	glass bottles with polymer-lined metal lids	33.33 (1/3)	ND-94.43
	all-glass bottle	0 (0/1)	ND

^a^: No detection.

**Table 10:** Detection of MeP and PrP in honey with different packaging materials.

	Packaging Material	Detection Rate (%)	Range (μg/kg)
MeP	PET	82.14 (23/28)	ND ^a^-439.5
	PP	100 (3/3)	37.2–61.3
	laminated polymer/foil pouches	80 (4/5)	ND-320.9
	glass bottles with plastic lids	71.43 (5/7)	ND-176.9
	glass bottles with polymer-lined metal lids	100 (3/3)	64.86–299.2
	all-glass bottle	100 (1/1)	179.8
PrP	PET	85.71 (24/28)	ND-136.7
	PP	66.67 (2/3)	ND- < LOQ ^b^
	laminated polymer/foil pouches	80 (4/5)	ND-45.12
	glass bottles with plastic lids	71.42 (5/7)	ND-109.3
	glass bottles with polymer-lined metal lids	100 (3/3)	ND- < LOQ
	all-glass bottle	0 (0/1)	ND

^a^: No detection; ^b^: Below the LOQ.

**Table 11:** Detection of 6 typical EDCs in honey at home and abroad.

Place of Origin	EDCs	Detection Rate (%)	Range (μg/kg)	Geometric Mean Concentration (μg/kg)
China	TCS	27.5 (11/40)	ND ^a^ 144.6	3.14
	TCC	20.0 (8/40)	ND- < LOQ ^b^	1.44
	MTCS	0 (0/40)	ND	ND
	BPF	97.5 (39/40)	ND-642.4	144.9
	4HBP	32.5 (13/40)	ND-294.9	3.32
	MeP	82.5 (33/40)	ND-439.5	27.39
	PrP	85.0 (34/40)	ND-120.3	10
Abroad	TCS	42.86 (3/7)	ND- < LOQ	5.57
	TCC	14.28 (1/7)	ND- < LOQ	1.34
	MTCS	0 (0/7)	ND	ND
	BPF	100 (7/7)	187.6–1193	527.1
	4HBP	57.14 (4/7)	ND-99.32	12.89
	MeP	85.71 (6/7)	ND-320.9	56.19
	PrP	57.14 (4/7)	ND-136.7	17.80

^a^: No detection; ^b^: Below the LOQ.

## Data Availability

The original contributions presented in the study are included in the article/Supplementary Material, further inquiries can be directed to the corresponding author/s.

## Supplementary Materials

The following supporting information can be downloaded at https://www.mdpi.com/article/10.3390/foods14122006/s1, Table S1: Detection of 7 typical endocrine disruptors in acacia honey; Table S2: Detection of 7 typical endocrine disruptors in rape flower honey and jujube honey; Table S3: Detection of 7 typical endocrine disruptors in linden honey; Table S4: Detection of 7 typical endocrine disruptors in citrus, wolfberry, vitex, sunflower, loquat, motherwort, and milk vetch honey; Table S5: Detection of 7 typical endocrine disruptors in multifloral honey; Table S6: Detection of 7 typical endocrine disruptors in PP-packed, laminated polymer/foil pouch-packed honey and glass bottles with polymer-lined metal lid-packed honey; Table S7: Detection of 7 typical endocrine disruptors in glass bottle-packed honey and glass bottles with plastic lid-packed honey; Table S8: Detection of 7 typical endocrine disruptors in PET-packed honey; Table S9: Contamination of seven typical endocrine disruptors in honey samples from China; Table S10: Contamination of seven typical endocrine disruptors in honey samples from abroad.

## References

[1] He K., Chen R., Xu S., Ding Y., Wu Z., Bao M., He B., Li S. (2024). Environmental endocrine disruptor-induced mitochondrial dysfunction: A potential mechanism underlying diabetes and its complications. Front. Endocrinol..

[2] Kloas W., Stöck M., Lutz I., Ziková-Kloas A. (2024). Endocrine disruption in teleosts and amphibians is mediated by anthropogenic and natural environmental factors: Implications for risk assessment. Philos. Trans. R. Soc. B.

[3] Dalamaga M., Kounatidis D., Tsilingiris D., Vallianou N.G., Karampela I., Psallida S., Papavassiliou A.G. (2024). The Role of Endocrine Disruptors Bisphenols and Phthalates in Obesity: Current Evidence, Perspectives and Controversies. Int. J. Mol. Sci..

[4] Bocato M.Z., Cesila C.A., Lataro B.F., de Oliveira A.R.M., Campíglia A.D., Barbosa F. (2020). A fast-multiclass method for the determination of 21 endocrine disruptors in human urine by using vortex-assisted dispersive liquid-liquid microextraction (VADLLME) and LC-MS/MS. Environ. Res..

[5] Gee R.H., Charles A., Taylor N., Darbre P.D. (2008). Oestrogenic and androgenic activity of triclosan in breast cancer cells. J. Appl. Toxicol..

[6] Fang J.L., Stingley R.L., Beland F.A., Harrouk W., Lumpkins D.L., Howard P. (2010). Occurrence, efficacy, metabolism, and toxicity of triclosan. J. Environ. Sci. Health Part C.

[7] Geer L.A., Pycke B.F., Waxenbaum J., Sherer D.M., Abulafia O., Halden R.U. (2017). Association of birth outcomes with fetal exposure to parabens, triclosan and triclocarban in an immigrant population in Brooklyn, New York. J. Hazard. Mater..

[8] Higashihara N., Shiraishi K., Miyata K., Oshima Y., Minobe Y., Yamasaki K. (2007). Subacute oral toxicity study of bisphenol F based on the draft protocol for the “Enhanced OECD Test Guideline no. 407”. Arch. Toxicol..

[9] Xu G., Huang M., Hu J., Liu S., Yang M. (2024). Bisphenol A and its structural analogues exhibit differential potential to induce mitochondrial dysfunction and apoptosis in human granulosa cells. Food Chem. Toxicol..

[10] Cheng S., Huang M., Liu S., Yang M. (2024). Bisphenol F and bisphenol S induce metabolic perturbations in human ovarian granulosa cells. Arab. J. Chem..

[11] Choi S.-I., Kwon H.-Y., Han X., Men X., Choi Y.-E., Jang G.-W., Park K.-T., Han J., Lee O.-H. (2021). Environmental obesogens (bisphenols, phthalates and parabens) and their impacts on adipogenic transcription factors in the absence of dexamethasone in 3T3-L1 cells. J. Steroid Biochem. Mol. Biol..

[12] Witorsch R.J., Thomas J.A. (2010). Personal care products and endocrine disruption: A critical review of the literature. Crit. Rev. Toxicol..

[13] Vo T.T., Yoo Y.M., Choi K.C., Jeung E.B. (2010). Potential estrogenic effect(s) of parabens at the prepubertal stage of a postnatal female rat model. Reprod. Toxicol..

[14] Chen J., Ahn K.C., Gee N.A., Gee S.J., Hammock B.D., Lasley B.L. (2007). Antiandrogenic properties of parabens and other phenolic containing small molecules in personal care products. Toxicol. Appl. Pharmacol..

[15] Shariatifar N., Dadgar M., Fakhri Y., Shahsavari S., Moazzen M., Ahmadloo M., Kiani A., Aeenehvand S., Nazmara S., Khanegah A.M. (2020). Levels of polycyclic aromatic hydrocarbons in milk and milk powder samples and their likely risk assessment in Iranian population. J. Food Compos. Anal..

[16] Wang X., Wang M., Wang X., Du L., Chu F., Ding W., Gu H., Wang H. (2018). A novel naphthalene carboxylic acid-based ionic liquid mixed disperser combined with ultrasonic-enhanced in-situ metathesis reaction for preconcentration of triclosan and methyltriclosan in milk and eggs. Ultrason. Sonochemistry.

[17] Yao L., Lv Y.-Z., Zhang L.-J., Liu W.-R., Zhao J.-L., Liu Y.-S., Zhang Q.-Q., Ying G.-G. (2018). Determination of 24 personal care products in fish bile using hybrid solvent precipitation and dispersive solid phase extraction cleanup with ultrahigh performance liquid chromatography-tandem mass spectrometry and gas chromatography-mass spectrometry. J. Chromatogr. A.

[18] Wu H., Wu L.-H., Wang F., Gao C.-J., Chen D., Guo Y. (2019). Several environmental endocrine disruptors in beverages from South China: Occurrence and human exposure. Environ. Sci. Pollut. Res..

[19] Zapata N.I., Peñuela G.A. (2021). Modified QuEChERS/UPLC-MS/MS method to monitor triclosan, ibuprofen, and diclofenac in fish *Pseudoplatystoma magdaleniatum*. Food Anal. Methods.

[20] Gentili A., Marchese S., Perret D. (2008). MS techniques for analyzing phenols, their metabolites and transformation products of environmental interest. TrAC Trends Anal. Chem..

[21] Manav Ö.G., Dinç-Zor Ş., Alpdoğan G. (2019). Optimization of a modified QuEChERS method by means of experimental design for multiresidue determination of pesticides in milk and dairy products by GC–MS. Microchem. J..

[22] Bemrah N., Jean J., Rivière G., Sanaa M., Leconte S., Bachelot M., Deceuninck Y., Le Bizec B., Dauchy X., Roudot A.-C. (2014). Assessment of dietary exposure to bisphenol A in the French population with a special focus on risk characterisation for pregnant French women. Food Chem. Toxicol..

[23] Yang G., Tang Y., Liu X., Wang L., Qin L., Li D., Shen X., Kong C., Zhai W., Fodjo E.K. (2024). Determination of Free Glycidol and Total Free Monochloropropanediol in Fish and Krill Oil with Simple Aqueous Derivatization and High-Performance Liquid Chromatography-Tandem Mass Spectrometry. Foods.

[24] Wang K., Meng X., Yan X., Fan K. (2024). Nanozyme-based point-of-care testing: Revolutionizing environmental pollutant detection with high efficiency and low cost. Nano Today.

[25] Zheng J., Kuang Y., Zhou S., Gong X., Ouyang G. (2023). Latest Improvements and Expanding Applications of Solid-Phase Microextraction. Anal. Chem..

[26] Guo W., Tao H., Tao H., Shuai Q., Huang L. (2024). Recent progress of covalent organic frameworks as attractive materials for solid-phase microextraction: A review. Anal. Chim. Acta.

[27] Baghaei P.A.M., Mogaddam M.R.A., Farajzadeh M.A., Mohebbi A., Sorouraddin S.M. (2023). Application of deep eutectic solvent functionalized cobalt ferrite nanoparticles in dispersive micro solid phase extraction of some heavy metals from aqueous samples prior to ICP-OES. J. Food Compos. Anal..

[28] Huo S., Deng X., Yang N., Qin M., Zhang X., Yao X., An C., Zhou P., Lu X. (2024). A durable hydrophobicity hydrazone covalent organic framework coating for solid phase microextraction of polycyclic aromatic hydrocarbons in food and environmental sample. Chem. Eng. J..

[29] Chirani M.R., Kowsari E., Teymourian T., Ramakrishna S. (2021). Environmental impact of increased soap consumption during COVID-19 pandemic: Biodegradable soap production and sustainable packaging. Sci. Total. Environ..

[30] Sidor A., Rzymski P. (2020). Dietary Choices and Habits during COVID-19 Lockdown: Experience from Poland. Nutrients.

[31] Zhang Y., Li H., Wu H., Zong X., Zhu Z., Pan Y., Li J., Zheng X., Wei M., Tong M. (2017). The 5th national survey on the physical growth and development of children in the nine cities of China: Anthropometric measurements of Chinese children under 7 years in 2015. Am. J. Phys. Anthropol..

[32] Environmental Protection Agency (2005). Human Health Risk Assessment Protocol, Chapter 7: Characterizing Risk and Hazard.

[33] European Food Safety Authority (EFSA) (2004). Opinion of the Scientific Panel on food additives flavourings processing aids materials in contact with food (AFC) related to para hydroxybenzoates (E214–219). EFSA J..

[34] Minnesota Department of Health (2017). Incoporation of human equivalent dose calculations into derivation of oral reference doses. MDH Health Risk Assessment Methods.

[35] Environmental Protection Agency (2011). Recommendations for and Documentation of Biological Values for Use in Risk Assessment.

[36] Minnesota Department of Health (2015). Toxicological summary for triclosan. Human Health-Based Water Guidance Table.

[37] Minnesota Department of Health (2013). Toxicological summary for triclocarban. Human Health-Based Water Guidance Table.

[38] Chen M.L., Chen C.H., Huang Y.F., Chen H.C., Chang J.W. (2022). Cumulative Dietary Risk Assessment of Benzophenone-Type Photoinitiators from Packaged Foodstuffs. Foods.

[39] Boberg J., Taxvig C., Christiansen S., Hass U. (2010). Possible endocrine disrupting effects of parabens and their metabolites. Reprod. Toxicol..

[40] Lemini C., Jaimez R., Ávila M.E., Franco Y., Larrea F., Lemus A.E. (2003). In vivo and in vitro estrogen bioactivities of alkyl parabens. Toxicol. Ind. Health.

[41] Wei F., Mortimer M., Cheng H., Sang N., Guo L.-H. (2021). Parabens as chemicals of emerging concern in the environment and humans: A review. Sci. Total. Environ..

[42] Bereketoglu C., Pradhan A. (2019). Comparative transcriptional analysis of methylparaben and propylparaben in zebrafish. Sci. Total. Environ..

[43] Li M., Zhou S., Wu Y., Li Y., Yan W., Guo Q., Xi Y., Chen Y., Li Y., Wu M. (2021). Prenatal exposure to propylparaben at human-relevant doses accelerates ovarian aging in adult mice. Environ. Pollut..

[44] Tan J., Kuang H., Wang C., Liu J., Pang Q., Xie Q., Fan R. (2021). Human exposure and health risk assessment of an increasingly used antibacterial alternative in personal care products: Chloroxylenol. Sci. Total. Environ..

[45] Freire C., Molina-Molina J.-M., Iribarne-Durán L.M., Jiménez-Díaz I., Vela-Soria F., Mustieles V., Arrebola J.P., Fernández M.F., Artacho-Cordón F., Olea N. (2019). Concentrations of bisphenol A and parabens in socks for infants and young children in Spain and their hormone-like activities. Environ. Int..

