# Supplementary material for: Distribution and Health Risk Assessment of Triclosan and Other Typical Endocrine Disruptors in Honey
Source: Foods. 2025 Jun 6;14(12):2006. doi: 10.3390/foods14122006 (PMC12191833; doi:10.3390/foods14122006)
Supplement: Supplementary file 1 [file foods-14-02006-s001.zip › foods-3530607-supplementary.pdf]

Supporting Information for

Distribution and Health Risk Assessment of Triclosan and Other Typical Endocrine Disruptors in Honey

Jianing Wang, Meiqi Gao, Hongmei Li, Xinyan Hou, Aijun Gong and Yanqiu Cao

Table captions

Table S1 Detection of 7 typical endocrine disruptors in acacia honey.

Table S2 Detection of 7 typical endocrine disruptors in rape flower honey and jujube honey.

Table S3 Detection of 7 typical endocrine disruptors in linden honey.

Table S4 Detection of 7 typical endocrine disruptors in citrus, wolfberry, vitex, sunflower, loquat, motherwort, and milk vetch honey.

Table S5 Detection of 7 typical endocrine disruptors in multifloral honey.

Table S6 Detection of 7 typical endocrine disruptors in PP-packed, laminated polymer/foil pouch-packed honey and glass bottles with polymer-lined metal lid-packed honey.

Table S7 Detection of 7 typical endocrine disruptors in glass bottle-packed honey and glass bottles with plastic lid-packed honey.

Table S8 Detection of 7 typical endocrine disruptors in PET-packed honey.

Table S9 Contamination of seven typical endocrine disruptors in honey samples from China.

Table S10 Contamination of seven typical endocrine disruptors in honey samples from abroad.

Table S1 Detection of 7 typical endocrine disruptors in acacia honey.

	Detected concentration (μg/kg)							
	Sample	TCS	TCC	MTCS	BPF	4HBP	MeP	PrP
acacia honey	1	ND[a]	ND	ND	<LOQ	ND	<LOQ	<LOQ
	Relative standard deviation (%)	-	-	-	-	-	-	-
	2	<LOQ[b]	<LOQ	ND	ND	ND	131.7	<LOQ
	Relative standard deviation (%)	-	-	-	-	-	1.7	-
	3	<LOQ	<LOQ	ND	<LOQ	ND	61.3	<LOQ
	Relative standard deviation (%)	-	-	-	-	-	1.0	-
	4	ND	ND	ND	<LOQ	50.62	37.2	<LOQ
	Relative standard deviation (%)	-	-	-	-	1.3	1.2	-
	5	ND	<LOQ	ND	<LOQ	ND	<LOQ	<LOQ
	Relative standard deviation (%)	-	-	-	-	-	-	-
	6	ND	ND	ND	469	ND	55.73	<LOQ
	Relative standard deviation (%)	-	-	-	1.6	-	1.5	-
	7	<LOQ	<LOQ	ND	<LOQ	ND	122.4	<LOQ
	Relative standard deviation (%)	-	-	-	-	-	1.7	-
	8	ND	ND	ND	<LOQ	ND	<LOQ	<LOQ
	Relative standard deviation (%)	-	-	-	-	-	-	-
	9	<LOQ	ND	ND	612.5	50.6	176.9	109.3
	Relative standard deviation (%)	-	-	-	1.9	1.2	2.4	2.6

a: no detection; b: below the LOQ.

Table S2 Detection of 7 typical endocrine disruptors in rape flower honey and jujube honey.

Nectar source	Detected concentration (μg/kg)							
	sample	TCS	TCC	MTCS	BPF	4HBP	MeP	PrP
rape flower honey	1	ND[a]	ND	ND	261.4	ND	149.5	ND
	Relative standard deviation (%)	-	-	-	0.8	-	2.2	-
	2	121	ND	ND	<LOQ[b]	<LOQ	114.9	<LOQ
	Relative standard deviation (%)	1.2	-	-	1.2	-	0.6	-
	3	ND	ND	ND	297.9	ND	<LOQ	<LOQ
	Relative standard deviation (%)	-	-	-	1.9	-	-	-
jujube honey	1	<LOQ	<LOQ	ND	241.9	ND	ND	<LOQ
	Relative	-	-	-	1.8	-	-	-

standard deviation (%)							
2	<LOQ	ND	ND	232.1	ND	51.42	ND
Relative standard deviation (%)	-	-	-	1.8	-	1.7	-
3	ND	ND	ND	642.4	<LOQ	ND	<LOQ
Relative standard deviation (%)	-	-	-	0.7	-	-	-
4	ND	ND	ND	479.8	ND	64.86	<LOQ
Relative standard deviation (%)	-	-	-	1.2	-	2.3	-

a: no detection; b: below the LOQ.

Table S3 Detection of 7 typical endocrine disruptors in linden honey.

	Detected concentration（μg/kg）							
	Sample	TCS	TCC	MTCS	BPF	4HBP	MeP	PrP
linden flower	1	ND[a]	ND	ND	<LOQ	ND	ND	<LOQ
	Relative standard deviation (%)	-	-	-	-	-	-	-
	2	144.6	ND	ND	170.4	ND	249.7	<LOQ
	Relative standard deviation (%)	1.9	-	-	1.2	-	1.3	-
	3	ND	ND	ND	593.7	ND	76.55	<LOQ
	Relative standard deviation (%)	-	-	-	1.7	-	1.1	-
	4	<LOQ[b]	ND	ND	234.5	ND	179.8	ND
	Relative standard deviation (%)	-	-	-	1.2	-	1.4	-

a: no detection; b: Below the LOQ.

Table S4 Detection of 7 typical endocrine disruptors in citrus, wolfberry, vitex, sunflower, loquat, motherwort, and milk vetch honey.

Nectar source	Detected concentration（μg/kg）							
	sample	TCS	TCC	MTCS	BPF	4HBP	MeP	PrP
citrus honey	1	<LOQ[b]	ND	ND	295.5	ND	89.65	56.86
	Relative standard deviation (%)	-	-	-	0.9	-	2.0	2.1
	2	ND[a]	ND	ND	376.7	ND	195.4	48.01
	Relative standard deviation (%)	-	-	-	1.2	-	0.6	1.9
	3	ND	ND	ND	190.7	94.43	299.2	<LOQ
	Relative standard	-	-	-	1.9	1.2	1.8	-

	deviation (%)							
wolfberry honey	1	<LOQ	ND	ND	<LOQ	<LOQ	54.34	<LOQ
	Relative standard deviation (%)	-	-	-	-	-	1.2	-
	2	ND	<LOQ	ND	ND	ND	72.19	120.3
	Relative standard deviation (%)	-	-	-	-	-	0.9	1.0
vitex honey	1	ND	ND	ND	224.7	ND	<LOQ	<LOQ
	Relative standard deviation (%)	-	-	-	1.7	-	-	-
	2	ND	ND	ND	254.1	ND	<LOQ	<LOQ
	Relative standard deviation (%)	-	-	-	1.2	-	-	-
	3	ND	ND	ND	415.2	172.3	<LOQ	<LOQ
	Relative standard deviation (%)	-	-	-	1.0	1.2	-	-
sunflower honey	1	ND	<LOQ	ND	154.6	ND	40.52	39.42
	Relative standard deviation (%)	-	-	-	1.7	-	1.6	1.2
	2	ND	ND	ND	189	ND	70.02	<LOQ
	Relative standard deviation (%)	-	-	-	1.4	-	0.7	-
	3	ND	ND	ND	<LOQ	<LOQ	ND	<LOQ
	Relative standard deviation (%)	-	-	-	-	-	-	-
loquat honey	1	ND	ND	ND	150.8	ND	<LOQ	<LOQ
	Relative standard deviation (%)	-	-	-	1.2	-	-	-
motherwort honey	1	ND	<LOQ	ND	154.6	ND	40.52	<LOQ
	Relative standard deviation (%)	-	-	-	0.9	-	1.4	-
milk vetch honey	1	ND	ND	ND	578.2	ND	439.5	<LOQ
	Relative standard deviation (%)	-	-	-	1.2	-	1.3	-

a: no detection; b: below the LOQ.

Table S5 Detection of 7 typical endocrine disruptors in multifloral honey.

	Detected concentration (μg/kg)							
	Sample	TCS	TCC	MTCS	BPF	4HBP	MeP	PrP
multifloral honey	1	ND[a]	ND	ND	199.3	ND	42.07	ND
	Relative standard deviation (%)	-	-	-	0.7	-	2.2	-

2	ND	ND	ND	315	52.59	84.11	ND
Relative standard deviation (%)	-	-	-	1.2	1.9	1.9	-
3	<LOQ[b]	ND	ND	346.2	<LOQ	194.9	<LOQ
Relative standard deviation (%)	-	-	-	1.7	-	1.4	-
4	ND	ND	ND	157.9	294.9	<LOQ	<LOQ
Relative standard deviation (%)	-	-	-	1.3	1.3	-	-
5	ND	<LOQ	ND	347.6	ND	ND	45.12
Relative standard deviation (%)	-	-	-	1.2	-	-	1.0
6	ND	ND	ND	426.8	72.37	82.01	ND
Relative standard deviation (%)	-	-	-	1.8	1.0	1.7	-
7	ND	ND	ND	<LOQ	ND	57.91	<LOQ
Relative standard deviation (%)	-	-	-	-	-	1.8	-
8	ND	ND	ND	1096	ND	ND	46.46
Relative standard deviation (%)	-	-	-	1.0	-	-	1.9
9	ND	<LOQ	ND	552.9	99.32	193.8	<LOQ
Relative standard deviation (%)	-	-	-	1.7	2.1	1.2	-
10	<LOQ	ND	ND	187.6	<LOQ	89.44	74.16
Relative standard deviation (%)	-	-	-	1.4	-	0.9	1.8
11	ND	ND	ND	1193	<LOQ	<LOQ	136.7
Relative standard deviation (%)	-	-	-	1.8	-	-	0.9
12	ND	ND	ND	580.4	52.52	320.9	ND
Relative standard deviation (%)	-	-	-	2.1	1.1	0.7	-
13	ND	ND	ND	290.7	63.67	ND	<LOQ
Relative standard deviation (%)	-	-	-	1.2	1.9	-	-

a: no detection; b: below the LOQ.

Table S6 Detection of 7 typical endocrine disruptors in PP-packed, laminated polymer/foil pouch-packed honey and glass bottles with polymer-lined metal lid-packed honey.

Packaging	Detected concentration（μg/kg）

Materials	Sample	TCS	TCC	MTCS	BPF	4HBP	MeP	PrP
PP	1	ND[a]	ND	ND	199.3	ND	42.07	ND
	Relative standard deviation (%)	-	-	-	0.9	-	2.7	-
	2	<LOQ[b]	<LOQ	ND	<LOQ	ND	61.3	<LOQ
	Relative standard deviation (%)	-	-	-	-	-	0.6	-
	3	ND	ND	ND	<LOQ	50.62	37.2	<LOQ
	Relative standard deviation (%)	-	-	-	-	0.9	0.8	-
laminated polymer/foil pouches	1	ND	ND	ND	157.9	294.9	<LOQ	<LOQ
	Relative standard deviation (%)	-	-	-	1.9	0.6	-	-
	2	ND	<LOQ	ND	347.6	ND	ND	45.12
	Relative standard deviation (%)	-	-	-	2.3	-	-	2.3
	3	ND	ND	ND	<LOQ	ND	<LOQ	<LOQ
	Relative standard deviation (%)	-	-	-	-	-	-	-
	4	ND	ND	ND	<LOQ	ND	57.91	<LOQ
	Relative standard deviation (%)	-	-	-	-	-	0.9	-
	5	ND	ND	ND	580.4	ND	320.9	ND
	Relative standard deviation (%)	-	-	-	1.8	-	1.1	-
glass bottles with polymer-lined metal lids	1	ND	ND	ND	190.7	94.43	299.2	<LOQ
	Relative standard deviation (%)	-	-	-	2.1	2.1	1.9	-
	2	<LOQ	<LOQ	ND	<LOQ	ND	122.4	<LOQ
	Relative standard deviation (%)	-	-	-	-	-	1.4	-
	3	ND	ND	ND	479.8	ND	64.86	<LOQ
	Relative standard deviation (%)	-	-	-	1.8	-	2.2	-

a: no detection; b: below the LOQ.

Table S7 Detection of 7 typical endocrine disruptors in glass bottle-packed honey and glass bottles with plastic lid-packed honey.

Packaging materials	Detected concentration（μg/kg）							
	Sample	TCS	TCC	MTCS	BPF	4HBP	MeP	PrP
glass bottles	1	ND[a]	ND	ND	290.7	63.67	ND	<LOQ
	Relative	-	-	-	1.1	1.6	-	-

with plastic lids	standard deviation (%)							
	2	<LOQ[b]	<LOQ	ND	241.9	ND	ND	<LOQ
	Relative standard deviation (%)	-	-	-	1.3	-	-	-
	3	<LOQ	ND	ND	<LOQ	<LOQ	53.4	<LOQ
	Relative standard deviation (%)	-	-	-	-	-	0.8	-
	4	ND	ND	ND	<LOQ	ND	<LOQ	<LOQ
	Relative standard deviation (%)	-	-	-	-	-	-	-
	5	ND	ND	ND	<LOQ	ND	<LOQ	<LOQ
	Relative standard deviation (%)	-	-	-	-	-	-	-
	6	ND	ND	ND	254.1	72.37	82.01	ND
	Relative standard deviation (%)	-	-	-	1.5	2.6	2.1	-
	7	<LOQ	ND	ND	426.8	50.6	176.9	109.3
	Relative standard deviation (%)	-	-	-	1.8	1.4	0.9	2.8
all-glass bottle	1	<LOQ	ND	ND	612.5	ND	179.8	ND
	Relative standard deviation (%)	-	-	-	2.1	-	1.6	-

a: no detection; b: below the LOQ.

Table S8 Detection of 7 typical endocrine disruptors in PET-packed honey.

Packaging materials	Detected concentration (μg/kg)							
	sample	TCS	TCC	MTCS	BPF	4HBP	MeP	PrP
PET	1	ND[a]	ND	ND	<LOQ	<LOQ	ND	<LOQ
	Relative standard deviation (%)	-	-	-	-	-	-	-
	2	<LOQ[b]	<LOQ	ND	ND	ND	131.7	<LOQ
	Relative standard deviation (%)	-	-	-	-	-	2.1	-
	3	ND	ND	ND	<LOQ	ND	ND	<LOQ
	Relative standard deviation (%)	-	-	-	-	-	-	-
	4	ND	ND	ND	224.7	ND	<LOQ	<LOQ
	Relative standard deviation (%)	-	-	-	1.2	-	-	-
	5	<LOQ	ND	ND	232.1	ND	51.42	ND
	Relative	-	-	-	1.6	-	1.5	-

	Relative standard deviation (%)							
	6	<LOQ	ND	ND	295.5	ND	89.65	56.86
	Relative standard deviation (%)	-	-	-	1.7	-	2.1	0.7
	7	ND	ND	ND	261.4	ND	149.5	ND
	Relative standard deviation (%)	-	-	-	1.9	-	1.1	-
	8	ND	ND	ND	315	52.59	84.11	ND
	Relative standard deviation (%)	-	-	-	2.3	0.9	1.6	-
	9	ND	<LOQ	ND	<LOQ	ND	72.19	120.3
	Relative standard deviation (%)	-	-	-	-	-	1.9	1.9
	10	ND	ND	ND	580.2	ND	169.1	<LOQ
	Relative standard deviation (%)	-	-	-	2.3	-	1.3	-

（续上表）

Packaging materials	Detected concentration（μg/kg）							
	Sample	TCS	TCC	MTCS	BPF	4HBP	MeP	PrP
PET	11	ND[a]	ND	ND	469	ND	55.73	<LOQ
	Relative standard deviation (%)	-	-	-	1.9	-	1.3	-
	12	ND	ND	ND	642.4	<LOQ	ND	<LOQ
	Relative standard deviation (%)	-	-	-	1.4	-	-	-
	13	<LOQ[b]	ND	ND	346.2	<LOQ	194.9	<LOQ
	Relative standard deviation (%)	-	-	-	1.3	-	0.6	-
	14	ND	ND	ND	150.8	ND	<LOQ	<LOQ
	Relative standard deviation (%)	-	-	-	1.3	-	-	-
	15	ND	<LOQ	ND	154.6	ND	40.52	39.42
	Relative standard deviation (%)	-	-	-	1.0	-	0.6	0.4
	16	ND	ND	ND	578.2	ND	439.5	<LOQ
	Relative standard deviation (%)	-	-	-	1.7	-	2.1	-
	17	144.6	ND	ND	170.4	ND	249.7	<LOQ
	Relative standard deviation (%)	1.2	-	-	0.9	-	1.6	-
	18	121	ND	ND	<LOQ	<LOQ	114.9	<LOQ
	Relative	1.6	-	-	-	-	1.7	-

	standard deviation (%)							
	19	ND	ND	ND	593.7	ND	76.55	<LOQ
	Relative standard deviation (%)	-	-	-	2.3	-	1.9	-
	20	ND	ND	ND	415.2	172.3	<LOQ	<LOQ
	Relative standard deviation (%)	-	-	-	1.7	2.3	-	-

（续上表）

Packaging materials	Detected concentration（μg/kg）							
	Sample	TCS	TCC	MTCS	BPF	4HBP	MeP	PrP
PET	21	<LOQ[b]	ND	ND	376.7	ND	195.4	48.01
	Relative standard deviation (%)	-	-	-	2.0	-	1.3	0.9
	22	ND[a]	ND	ND	297.9	ND	ND	<LOQ
	Relative standard deviation (%)	-	-	-	1.9	-	-	-
	23	ND	ND	ND	189	ND	70.02	<LOQ
	Relative standard deviation (%)	-	-	-	0.6	-	0.6	-
	24	ND	ND	ND	<LOQ	<LOQ	ND	<LOQ
	Relative standard deviation (%)	-	-	-	-	-	-	-
	25	ND	ND	ND	1096	ND	ND	46.46
	Relative standard deviation (%)	-	-	-	1.1	-	-	0.4
	26	ND	<LOQ	ND	552.9	99.32	193.8	<LOQ
	Relative standard deviation (%)	-	-	-	1.9	1.7	2.2	-
	27	<LOQ	ND	ND	187.6	<LOQ	89.44	74.16
	Relative standard deviation (%)	-	-	-	0.8	-	1.9	1.6
	28	ND	ND	ND	1193	<LOQ	<LOQ	136.7
	Relative standard deviation (%)	-	-	-	1.7	-	-	0.7

a: no detection; b: below the LOQ.

Table S9 Contamination of seven typical endocrine disruptors in honey samples from China.

Place of origin	Detected concentration（μg/kg）							
	Sample	TCS	TCC	MTCS	BPF	4HBP	MeP	PrP
China　Beijing	1	ND[a]	ND	ND	42.07	ND	199.3	ND
	Relative	-	-	-	0.9	-	1.3	-

		standard deviation (%)							
		2	<LOQ[b]	<LOQ	ND	<LOQ	ND	61.3	<LOQ
		Relative standard deviation (%)	-	-	-	-	-	2.6	-
		3	ND	ND	ND	415.2	172.3	33.84	36.64
		Relative standard deviation (%)	-	-	-	0.4	2.6	1.4	0.6
		1	ND	ND	ND	<LOQ	63.67	290.7	<LOQ
		Relative standard deviation (%)	-	-	-	-	1.1	0.9	-
		2	ND	ND	ND	ND	<LOQ	<LOQ	<LOQ
		Relative standard deviation (%)	-	-	-	-	-	-	-
		3	ND	ND	ND	137.2	ND	35.13	31.15
		Relative standard deviation (%)	-	-	-	1.9	-	2.1	0.7
		4	ND	ND	ND	254.1	ND	<LOQ	<LOQ
	Heibei	Relative standard deviation (%)	-	-	-	1.9	-	-	-
		5	ND	ND	ND	224.7	ND	34.8	<LOQ
		Relative standard deviation (%)	-	-	-	2.1	-	1.0	-
		6	<LOQ	ND	ND	232.1	ND	51.42	ND
		Relative standard deviation (%)	-	-	-	1.7	-	1.8	-
		7	<LOQ	<LOQ	ND	241.9	ND	ND	<LOQ
		Relative standard deviation (%)	-	-	-	0.3	-	-	-

（续上表）

Place of origin		Detected concentration（μg/kg）							
		Sample	TCS	TCC	MTCS	BPF	4HBP	MeP	PrP
China	Jiangxi	1	<LOQ	ND	ND	<LOQ	ND	ND	<LOQ
		Relative standard deviation (%)	-	-	-	-	-	-	-

		2	<LOQ	ND	ND	346.2	<LOQ	194.9	<LOQ
		Relative standard deviation (%)	-	-	-	2.0	-	1.3	-
		3	ND	ND	ND	157.9	294.9	28.19	<LOQ
		Relative standard deviation (%)	-	-	-	1.5	2.9	0.7	-
	Hubei	1	<LOQ	ND	ND	295.5	ND	89.65	56.86
		Relative standard deviation (%)	-	-	-	1.5	-	0.9	0.8
		2	ND	ND	ND	190.7	94.43	299.2	31.13
		Relative standard deviation (%)	-	-	-	0.7	1.4	0.8	1.3
		3	ND	ND	ND	261.4	ND	149.5	ND
		Relative standard deviation (%)	-	-	-	1.8	-	2.1	-
		4	ND	ND	ND	315	52.59	84.11	ND
		Relative standard deviation (%)	-	-	-	1.9	0.9	1.1	-
		5	ND	<LOQ	ND	347.6	ND	ND	45.12
		Relative standard deviation (%)	-	-	-	2.2	-	-	1.5
		6	ND	<LOQ	ND	103.5	ND	72.19	120.3
		Relative standard deviation (%)	-	-	-	1.6	-	1.8	1.4
		7	ND	ND	ND	580.2	ND	169.1	<LOQ
		Relative standard deviation (%)	-	-	-	0.6	-	1.4	-

（续上表）

Place of origin		Detected concentration（μg/kg）							
		Sample	TCS	TCC	MTCS	BPF	4HBP	MeP	PrP
China	Guangxi	1	<LOQ	<LOQ	ND	131.7	ND	ND	<LOQ
		Relative standard deviation (%)	-	-	-	1.6	-	-	-
	Shandong	1	ND	ND	ND	469	ND	55.73	<LOQ
		Relative standard	-	-	-	2.4	-	1.3	-

		deviation (%)							
		2	ND	ND	ND	642.4	<LOQ	ND	<LOQ
		Relative standard deviation (%)	-	-	-	1.4	-	-	-
		3	ND	ND	ND	150.8	ND	32.82	<LOQ
		Relative standard deviation (%)	-	-	-	1.5	-	1.1	-
		4	ND	<LOQ	ND	154.6	ND	40.52	35.42
		Relative standard deviation (%)	-	-	-	0.9	-	0.9	1.3
		5	ND	ND	ND	578.2	ND	439.5	34.41
		Relative standard deviation (%)	-	-	-	1.2	-	2.1	1.9
	Shanghai	1	ND	ND	ND	<LOQ	50.62	37.2	37.96
		Relative standard deviation (%)	-	-	-	-	0.9	1.3	1.4
	Neimenggu	1	<LOQ	ND	ND	137.4	<LOQ	54.34	<LOQ
		Relative standard deviation (%)	-	-	-	2.1	-	1.9	-
	Fujian	1	<LOQ	<LOQ	ND	<LOQ	ND	122.4	<LOQ
		Relative standard deviation (%)	-	-	-	-	-	1.9	-
		2	ND	ND	ND	479.8	ND	64.86	<LOQ
		Relative standard deviation (%)	-	-	-	0.8	-	1.6	-

（续上表）

Place of origin		Detected concentration（μg/kg）							
		Sample	TCS	TCC	MTCS	BPF	4HBP	MeP	PrP
China	Heilongjiang	1	144.6	ND	ND	170.4	ND	249.7	28.85
		Relative standard deviation (%)	0.6	-	-	0.9	-	1.7	0.5
		2	ND	ND	ND	593.7	ND	76.55	<LOQ
		Relative standard deviation (%)	-	-	-	2.2	-	1.3	-
		3	121	ND	ND	<LOQ	<LOQ	114.9	<LOQ

	Relative standard deviation (%)	1.1	-	-	-	-	0.9	-
Hunan	1	<LOQ	ND	ND	376.7	ND	195.4	48.01
	Relative standard deviation (%)	-	-	-	1.7	-	1.4	0.8
Qinghai	1	ND	ND	ND	297.9	ND	<LOQ	<LOQ
	Relative standard deviation (%)	-	-	-	1.9	-	-	-
Sichuang	1	ND	ND	ND	<LOQ	ND	<LOQ	<LOQ
	Relative standard deviation (%)	-	-	-	-	-	-	-
Gansu	1	ND	ND	ND	189	ND	70.02	<LOQ
	Relative standard deviation (%)	-	-	-	1.9	-	1.4	-
Shanxi	1	ND	ND	ND	426.8	72.37	82.01	ND
	Relative standard deviation (%)	-	-	-	1.3	0.8	1.9	-

a: no detection; b: below the LOQ.

Table S10 Contamination of seven typical endocrine disruptors in honey samples from abroad.

Place of origin		Detected concentration（μg/kg）							
		Sample	TCS	TCC	MTCS	BPF	4HBP	MeP	PrP
Abroad	Heilongjiang	1	144.6	ND	ND	170.4	ND	249.7	28.85
		Relative standard deviation (%)	0.6	-	-	0.9	-	1.7	0.5
		2	ND	ND	ND	593.7	ND	76.55	<LOQ
		Relative standard deviation (%)	-	-	-	2.2	-	1.3	-
		3	121	ND	ND	<LOQ	<LOQ	114.9	<LOQ
		Relative standard deviation (%)	1.1	-	-	-	-	0.9	-
	Hunan	1	<LO	ND	ND	376.7	ND	195.4	48.01

		Q						
	Relative standard deviation (%)	-	-	-	1.7	-	1.4	0.8
Qinghai	1	ND	ND	ND	297.9	ND	<LOQ	<LOQ
	Relative standard deviation (%)	-	-	-	1.9	-	-	-
Sichuang	1	ND	ND	ND	<LOQ	ND	<LOQ	<LOQ
	Relative standard deviation (%)	-	-	-	-	-	-	-
Gansu	1	ND	ND	ND	189	ND	70.02	<LOQ
	Relative standard deviation (%)	-	-	-	1.9	-	1.4	-
Shanxi	1	ND	ND	ND	426.8	72.37	82.01	ND
	Relative standard deviation (%)	-	-	-	1.3	0.8	1.9	-

a: no detection; b: below the LOQ.
